# Strong and aversive cold processing and pain facilitation in fibromyalgia patients relates to augmented thermal grill illusion

**DOI:** 10.1038/s41598-023-42288-7

**Published:** 2023-09-25

**Authors:** Petra Bäumler, Anna Brenske, Andreas Winkelmann, Dominik Irnich, Beate Averbeck

**Affiliations:** 1 Multidisciplinary Pain Center, Department of Anaesthesiology, LMU University Hospital, LMU Munich, Munich, Germany; 2 Walter Brendel Center of Experimental Medicine (WBex), Biomedical Center Munich (BMC), LMU Munich, Großhaderner Str. 9, 82152 Planegg-Martinsried, Germany; 3 Department of Orthopaedics and Trauma Surgery, Musculoskeletal University Center Munich (MUM), LMU University Hospital, LMU Munich, Munich, Germany

**Keywords:** Diseases of the nervous system, Neural circuits, Sensory processing

## Abstract

The thermal grill illusion (TGI) is assumed to result from crosstalk between the thermoreceptive and nociceptive pathways. To elucidate this further, we compared 40 female fibromyalgia patients to 20 healthy women in an exploratory cross-sectional study. Sensations (cold, warm/heat, unpleasantness, pain and burning) evoked by 20 °C, 40 °C and alternating 20 °C/40 °C (TGI) and somatosensory profiles according to standardized quantitative sensory testing (QST) were assessed on the palm of the dominant hand. Compared to healthy controls, fibromyalgia patients reported stronger thermal grill-evoked cold, warm, unpleasantness and pain as well as stronger and more aversive 20 °C- and 40 °C-evoked sensations. They showed a loss in warm, mechanical and vibration detection, a gain in thermal pain thresholds and higher temporal summation (TS). Among QST parameters higher TS in fibromyalgia patients was most consistently associated with an augmented TGI. Independently, an increased TGI was linked to cold (20 °C) but less to warm (40 °C) perception. In fibromyalgia patients all thermal grill-evoked sensations were positively related to a higher 20 °C-evoked cold sensation and/or 20 °C-evoked unpleasantness. In conclusion, the TGI appears to be driven mainly by the cold-input. Aversive cold processing and central pain facilitation in fibromyalgia patients seem to independently augment the activation of the pain pathway.

## Introduction

The thermal grill illusion (TGI), first described by Thunberg in 1886, means a strong, not necessarily painful, but often unpleasant sensation of heat evoked by an interleaved application of innocuous cool and warm stimuli^1–4^. Parts of the contact area can also be perceived as cool^5^. This phenomenon is thought to be based on a crosstalk between central thermoreceptive and nociceptive pathways^6^, albeit mechanisms are still discussed controversially.

A widely discussed hypothesis proposes an unmasking of C-fiber driven input to the pain pathway, analogically to cold-evoked burning (disinhibition theory^7,8^). Several studies suggest that C-fiber input to the pain pathway is controlled by central mechanisms dependent on Aδ-cold fiber activity^9–12^. Regarding the TGI, Craig and Bushnell showed in cats that interleaved warm and cold stimuli cause a mismatch between inhibitory and excitatory input at spinal neurons responsive to noxious heat pinch and cold^13^. They also found that the thermal grill induced brain activation in humans resembles that of cold pain more than that of heat pain^14^. Later research complemented these findings by showing that innocuous cold activates Aδ-cold fibers and polymodal C-fibers which respond to cooling and warming^15,16^. In healthy volunteers, the TGI seems to be rather linked to the cold- than to the warm-input^17–20^. The stronger TGI in women has consequently been attributed to their higher cold sensitivity^18^. The disinhibition theory can explain the painful cold and heat sensations during the TGI, however, it does not explain the intense thermal grill-evoked warm sensation. As the induction of the TGI depends as much on the temperature of the warm stimuli as on the temperature of the cool stimuli, a convergence of thermal afferents has been proposed^21,22^. A population coding framework based on an interpolation of collective activity of populations of neurons was attempted to reconcile these two theories^6^.

To clarify TGI mechanisms further, investigations in patients exhibiting pronounced alterations in somatosensory function appear decisive^8^. To date, however, only few studies have evaluated the TGI in patient populations. In borderline disorder, major depression and schizophrenia reduced thermal grill-evoked sensations were observed in parallel with cold and heat hypoalgesia^23–25^. A mixed population of chronic pain patients with intact cold and warm processing showed a reduced TGI in comparison to healthy controls^26^. Fibromyalgia patients appear suitable to further investigate the crosstalk between thermoreceptive and nociceptive systems during the TGI, since patients show variable sensory sings linked to peripheral and central nervous system alterations^27,28^. A high variance in sensory parameters is specific to fibromyalgia patients rendering this population particularly suitable for association analyses. Fibromyalgia describes a complex of symptoms such as chronic widespread musculoskeletal pain, hyperalgesia at muscle and tendon insertions, headache, unrefreshed sleep, fatigue, cognitive dysfunction and depression^29–31^. More recently, small-fiber neuropathy was shown to play a role in fibromyalgia pain^32^. Thus, in the present exploratory cross-sectional study we investigated associations between thermal grill-evoked sensations and somatosensory function evaluated by standardized quantitative sensory testing (QST) in fibromyalgia patients. Alterations in thermal grill-evoked sensations and somatosensory function in fibromyalgia patients were determined by a comparison to a healthy control group.

## Results

### Participant characteristics

#### Clinical characteristics

**Figure 1:**
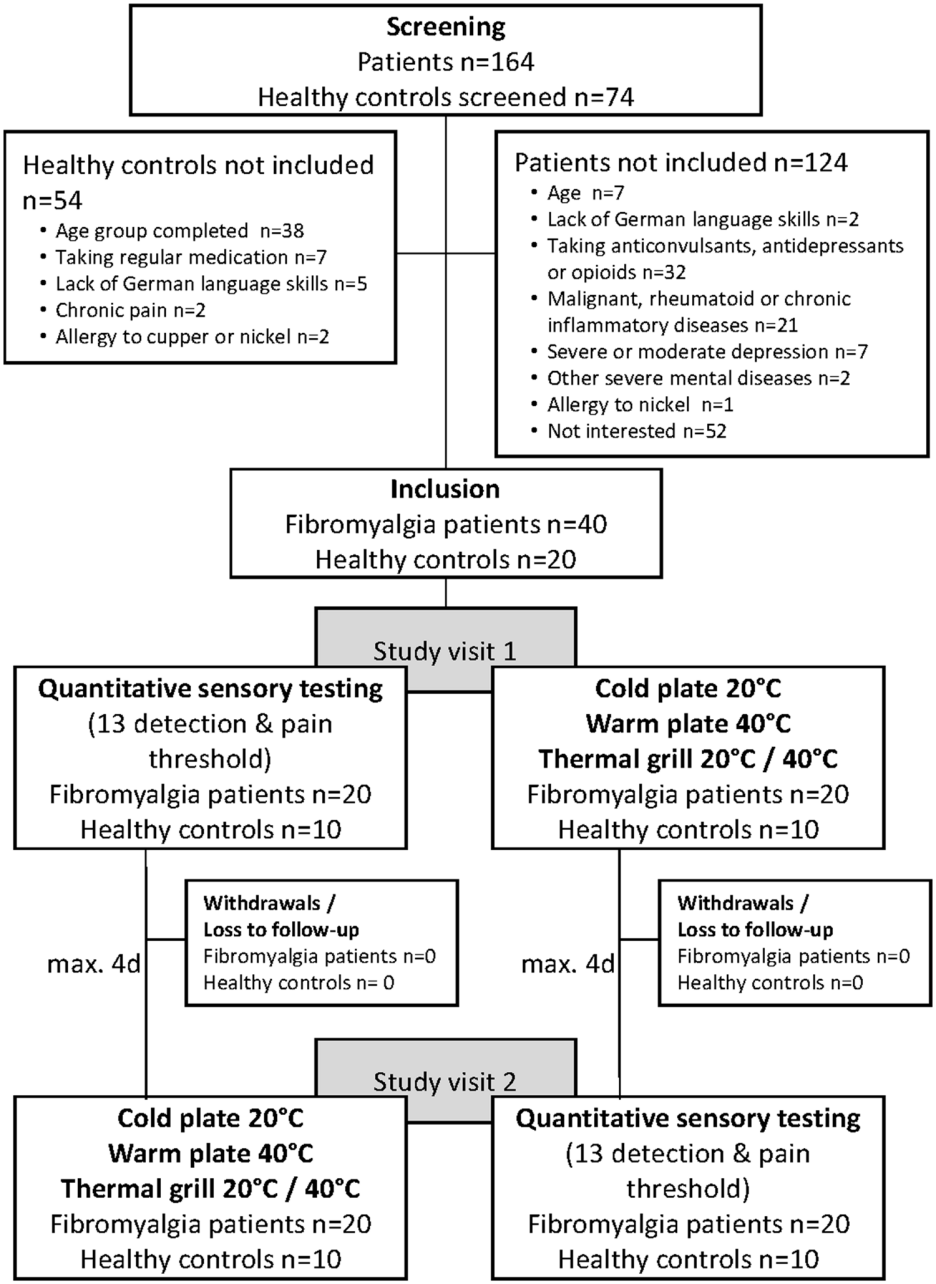
Study flow chart.

Forty of 164 screened female fibromyalgia patients and 20 of 74 screened healthy, female volunteers were included in this study (Fig. 1). There were neither losses to follow-up nor withdrawals from the study. Characteristics of patients and healthy controls are summarized in Table 1. The age distribution was similar between the two groups. On average, patients reported fibromyalgia associated pain for about ten years and a clinically relevant pain intensity during the last week before the first study visit. Over three quarters of the patients showed a pain chronification stage of III according to the MPSS. Three patients were taking analgesic medication on demand. According to the inclusion criteria, the maximum DASS score for depression was twelve in the fibromyalgia group and seven in the healthy control group. In comparison to healthy women, women with fibromyalgia showed significantly higher scores on all three DASS subscales; depression, anxiety and stress.

**Table 1:** Characteristics of fibromyalgia patients and healthy controls.

		Fibromyalgia patients n = 40		Healthy controls n = 20		*p*-value
Age years, md [IQR]		51.5 [45.3; 55.0]	21–60	50.0 [36.5; 54.8]	21–59	0.677
Pain duration months, md [IQR]		141.0 [52.0; 250.8]	13–613	–	–	–
Pain intensity NRS 0–10, md [IQR]		6.0 [4.3; 7.0]	3–10	–	–	–
DASS–depression 0–21, md [IQR]		5.0 [2.0; 6.0]	0–12	0.0 [0.0; 1.0]	0–7	< 0.001*
DASS–anxiety 0–21, md [IQR]		5.5 [2.0; 8.0]	0–13	0.5 [0.0; 1.8]	0–5	< 0.001*
DASS–stress 0–21, md [IQR]		8.5 [5.0; 12.8]	2–18	2.0 [0.0; 5.0]	0–13	< 0.001*
Age group, n (%)	21–30 years	5 (12.5)		3 (15.0)		0.795 expected frequencies < 5
	31–40 years	4 (10.0)		2 (10.0)		
	41–50 years	8 (20.0)		6 (30.0)		
	51–60 years	23 (57.5)		9 (45.0)		
MPSS, n (%)	I	1 (2.5)		–	–	–
	II	7 (17.5)		–	–	–
	III	32 (80.0)		–	–	–
Analgesic medication, n (%) ibuprofen / metamizole on demand		3 (7.5)	–		–	–

Group comparisons by Mann–Whitney-U Test (MW) for continuous data and by Chi-square tes for categorical data; *group differences significant on an α-level of 5%; NRS Numeric rating scale;, *DASS* Depression; anxiety and stress scales; *MPSS* Mainz pain staging system; *NSAID* Nonsteroidal anti-inflammatory drugs; *md* Median; *IQR* Interquartile range; *min* Minimum; *max* Maximum; *KW* Kruskal–Wallis test.

#### Sensory profile of fibromyalgia patients in comparison to healthy controls

**Figure 2:**
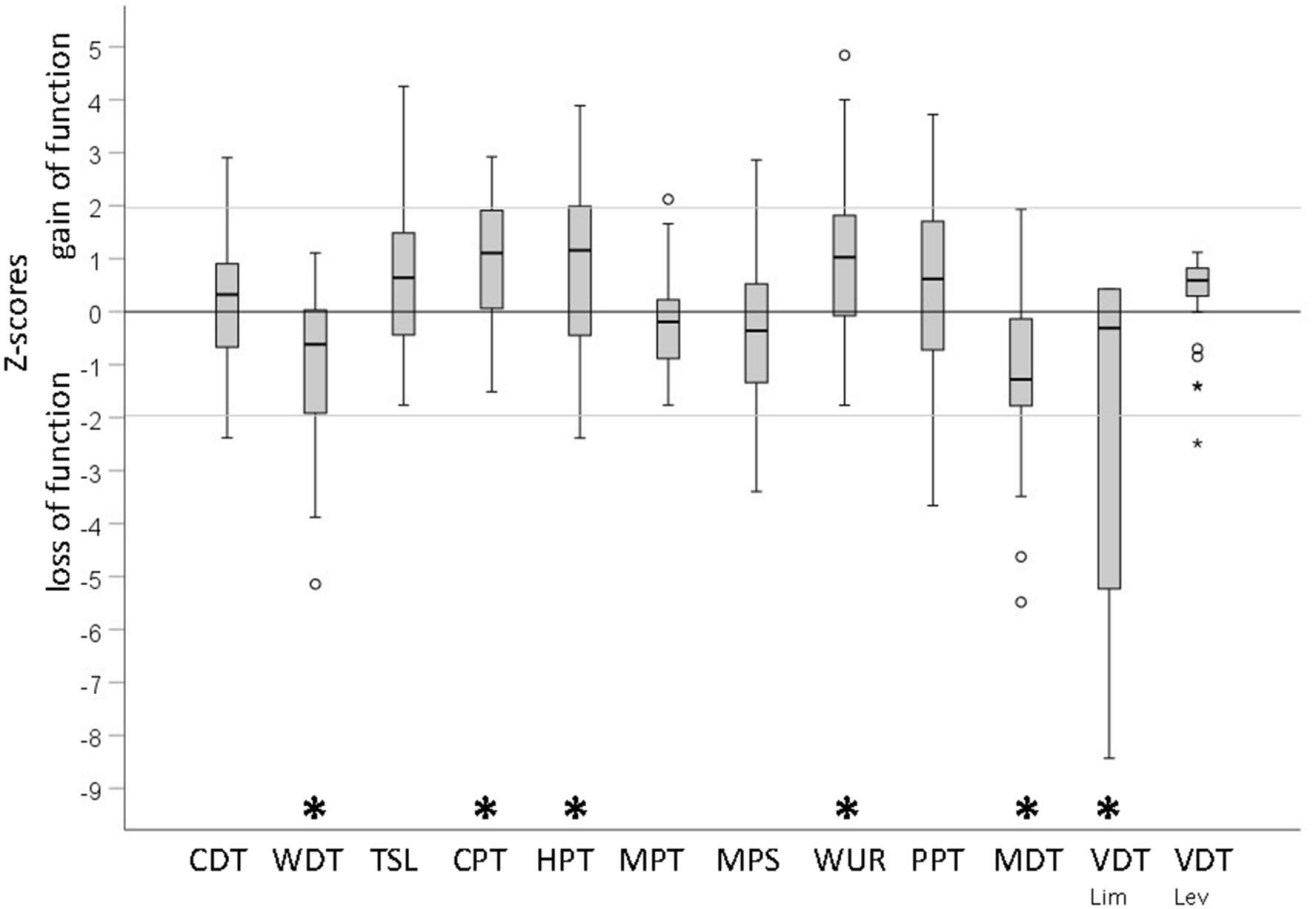
QST profile of Fibromyalgia patients. Z-Scores of sensory thresholds of fibromyalgia patients (n = 40) calculated with reference to the healthy control group (n = 20) with z = (x–mean(healthy))/SD(healthy); *CDT* Cold detection threshold; *WDT* Warm detection threshold; *TSL* Thermal sensory limen; *CPT* Cold pain threshold; *HPT* Heat pain threshold; *MDT* Mechanical detection threshold; *MPT* Mechanical pain threshold; *MPS* Mechanical pain sensitivity; *WUR* Wind-up ratio; *VDT*_Lim_ Vibration detection threshold by the method of limits (x/8); *VDT*_Lev_ Vibration detection threshold by the method of levels in microns; *PPT* Pressure pain threshold; *significant difference in comparison to the healthy control group on an α-level of 5%.

Sensory thresholds evaluated at the thenar eminence according to the standardized QST battery of the German Research Network on neuropathic pain (DFNS) revealed a characteristic sensory profile of fibromyalgia patients. In comparison to healthy controls, fibromyalgia patients showed significantly increased warm, mechanical and vibration detection thresholds (WDT, MDT, VDT), reduced cold and heat pain thresholds (CPT, HPT), significantly elevated pin-prick-evoked temporal summation of pain as quantified by the wind-up ratio (WUR) and significantly more frequent but low dynamic mechanical allodynia (DMA). Paradoxical heat sensation (PHS) evoked by cooling during the test of the thermal sensory limen was only observed in one woman suffering from fibromyalgia. (Table 2, Fig. 2).

**Table 2:** Descriptive statistics of sensory parameters.

Quantitative sensory testing		Fibromyalgia patients n = 40			Healthy controls n = 20			*p*-value				
		n (%)	md [IQR]	min–max	n (%)	md [IQR]	min–max					
CDT	Δ °C from 32°C		− 1.7	− 4– − 1		− 1.8	− 5– − 1	0.718				
			[−2.4; −1.4]			[−2.2; −1.5]						
WDT	Δ °C from 32°C		1.9	1–6		1.7	1–3	0.008*				
			[1.6; 2.7]			[1.4; 1.8]						
TSL	Δ °C CDT/WDT		3.0	1–6		3.5	2–7	0.060				
			[2.3; 4.3]			[2.8; 4.9]						
CPT	°C		17.3	0–29		9.1	0–24	0.003*				
			[9.9; 22.7]			[5.1; 14.2]						
HPT	°C		40.8	34–50		43.4	40–49	0.017*				
			[38.6; 45.0]			[41.4; 45.8]						
MPT	mN		45.3	8–147		39.5	11–169	0.384				
			[32.6; 77.5]			[20.1; 64.0]						
MPS	NRS (0–100)		1.7	0–36		2.0	0–14	0.380				
			[0.7; 4.0]			[1.2; 5.1]						
TS	(WUR)		3.1	1–14		2.1	1–6	0.015*				
			[1.9; 4.2]			[1.5; 2.5]						
PPT	kPa		295.9	160–690		349.9	249–523	0.215				
			[238.7; 389.1]			[278.0; 371.1]						
MDT	mN		0.6	0–5		0.3	0–1	0.006*				
			[0.3; 0.8]			[0.2; 0.5]						
VDT_Lim_	(x / 8)		7.9	7–8		8.0	8–8	0.001*				
			[7.2; 8.0]			[8.0; 8.0]						
VDT_Lev_	microns		1.0	0–7		1.8	0–7	0.178				
			[0.6; 1.6]			[0.6; 3.2]						
PHS	(x / 3)	1		0–1	–			1.000				
		(2.5)										
DMA	NRS (0–100)	9		0–1.9	–			0.023*				
		(22.5)										

Cold-, warm- and thermal grill-evoked sensations		Fibromyalgia patients n = 40					Healthy controls n = 20					*p*-value
		Total			With sensation		Total			With sensation		
		n (%)	md [IQR]	min–max	md [IQR]	min–max	n (%)	md [IQR]	min–max	md [IQR]	min–max	
Cold-evoked sensations (20°C)	Cold	40	31.7	10–70			20	23.3	8–33			0.003*
		(100)	[20.8; 48.7]				(100)	[13.3; 30.0]				
	Warm	4		0–7		3–7	–					0.147
		(10)										
	Unpleasant	26	10.0	0–50	16.7	3–50	1		0–6			< 0.001*
		(65)	[0; 21.3]		[10.0; 30.8]		(5)					
	Pain	10	0	0–36	13.3	3–36	–					0.016*
		(25)	[0; 2.5]		[3.3; 24.2]							
	Burning	8					1					0.249
		(20)					(5)					
Warm-evoked sensations (40°C)	Cold	1		0–12			–					0.480
		(3)										
	Warm	40	31.7	13–67			20	23.3	10–40			0.017*
		(100)	[23.3; 42.5]				(100)	[18.3; 32.5]				
	Unpleasant	12	0	0–47	15.8	3–47	1		0–5			0.024*
		(30)	[0; 5.0]		[5.4; 27.5]		(5)					
	Pain	3		0–20		7–20	–					0.213
		(8)										
	Burning	11					–					0.011*
		(28)										
Thermal grill-evoked sensations (20°C / 40°C)	Cold	39	40.0	0–77			20	20.0	7–42			0.001*
		(98)	[23.8; 56.7]				(100)	[12.1; 33.3]				
	Warm	40	55.0	23–83			20	35.8	13–50			< 0.001*
		(100)	[40.4; 64.6]				(100)	[27.9; 43.3]				
	Unpleasant	38	40.0	0–93			16	14.7	0–50	20.8	3–50	0.001*
		(95)	[23.3; 65.0]				(80)	[4.2; 26.7]		[13.3; 34.2]		
	Pain	25	13.3	0–80	26.7	2–80	4		0–13		1–13	<0.001*
		(63)	[0.0; 37.1]		[15.8; 45.8]		(20)					
	Burning	25					11					0.590
		(63)					(55)					

Group comparisons by Mann–Whitney-U Test (MW) for continuous data and by Fisher Test for dichotomous data,*group differences significant on an α-level of 5%.

*CDT* Cold detection threshold as change from 32 °C (Δ°C); *WDT* Warm detection threshold as change form 32°C (Δ°C); *TSL* Thermal sensory limen as temperature change between cold and warm detection (Δ°C CDT/WDT); *CPT* Cold pain threshold in °C; *HPT* Heat pain threshold in °C; *MDT* Mechanical detection threshold in millinewton (mN); *MPT* Mechanical pain threshold in mN; *MPS* Mechanical pain sensitivity on the numeric rating scale (NRS 0–100); *TS* Temporal summation as evaluated by the wind-up ratio (WUR); V*DT*_*Lim*_ Vibration detection threshold by the method of limits (x/8); *VDT*_*Lev*_ Vibration detection threshold by the method of levels in microns; *PPT* Pressure pain threshold in kilopascal (kPa); *PHS* Paradoxical heat sensations (x/3); *DMA* Dynamic mechanical allodynia; *md* Median; *IQR* Interquartile range; *min* Minimum; *max* Maximum; cold and warm sensations, unpleasantness and pain measured on a numeric rating scale (0 - 100); burning yes / no.

#### Thermal perception and thermal grill illusion of fibromyalgia patients in comparison to healthy controls

**Figure 3:**
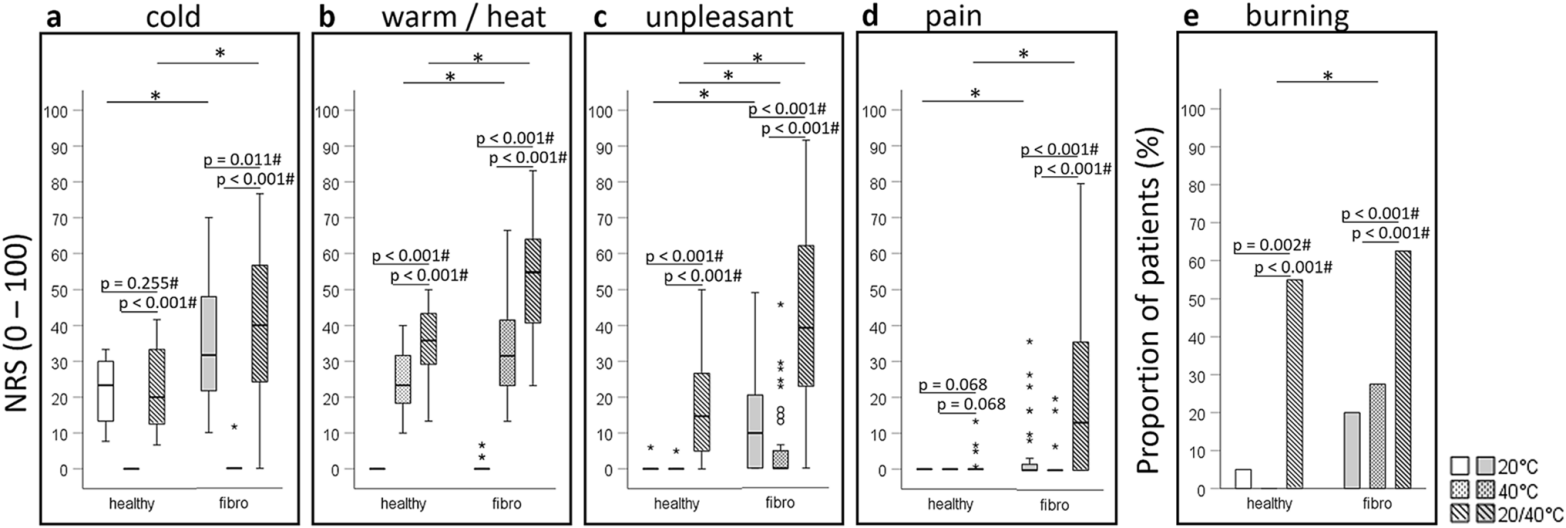
Sensations during contact with the cold and warm plate and under the thermal grill condition. (**a**, **b**, **c**, **d**) Box plots indicating median and interquartile range (IQR) as well as outliers (> 1.5 IQR) of the intensity of cold and warm/heat sensations, unpleasantness and pain on a numeric rating scale (NRS 0–100); statistical comparisons within groups by Wilcoxon rank-sum test and between groups by Mann–Whitney-U test; (**e**) Proportion of patients with a burning sensation—statistical comparison within groups by McNemar test and between groups by Fisher test; cold plate (20 °C): white boxes/bars, warm plate (40 °C): dotted boxes/bars, thermal grill condition (20 °C/40 °C): striped pattern boxes/bars; healthy: white shaded boxes/bars; fibromyalgia patients: grey shaded boxes/bars; *significant between group difference on an α-level of 5%; # significant within group difference on an α-level of 5%; *p*-values for group comparisons are displayed in Table2.

The cold sensation and unpleasantness during contact with the cold plate (20 °C) as well as the warm/heat sensation and unpleasantness during contact with the warm plate (40 °C) were rated significantly higher by fibromyalgia patients than by healthy controls. Additionally, unpleasantness, pain and burning during contact with the 20 °C- and 40 °C-thermal plates as well as paradoxical thermal sensations (20 °C-evoked warm and 40 °C-evoked cold) were almost exclusively restricted to fibromyalgia patients. During the thermal grill condition, fibromyalgia patients reported a significantly more intense cold and warm/heat sensation as well as significantly stronger unpleasantness and pain than healthy women. Frequencies of thermal grill-evoked burning were not significantly different between groups. (Table 2, Fig. 3).

**Figure 4:**
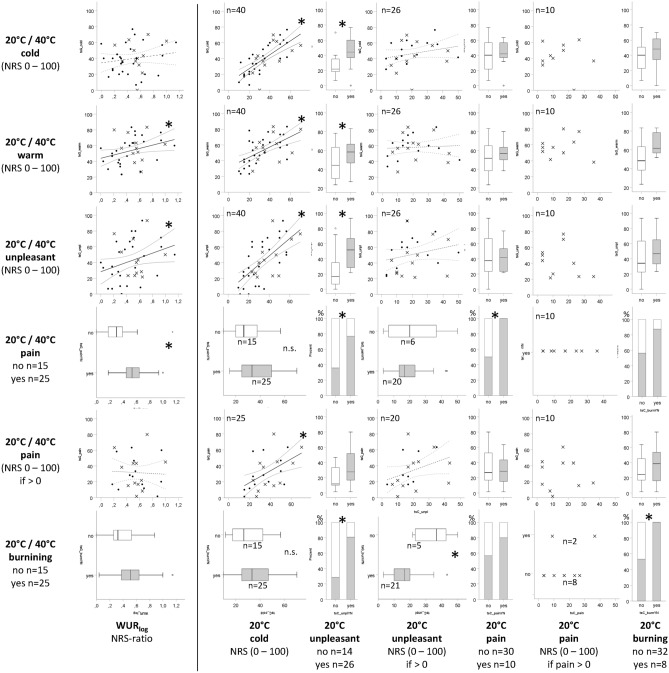
Associations of thermal grill (20 °C/40 °C)-evoked sensations with the temporal summation and cold (20 °C)-evoked sensations. Y-axes represent the thermal grill-evoked sensations and x-axes the log-transformed wind-up ratio (WUR_log_ quantifying temporal summation of pain, TS) and 20 °C-evoked sensations respectively. Scatter plots illustrate associations between continuous variables. Here, cases with 20 °C- and/or 40 °C-evoked pain are depicted as crosses and the remaining cases as points. Box-plots illustrate associations between a continuous and a dichotomous variable and bar plots associations between two dichotomous variables. Associations were evaluated by generalized linear models (GLM) or by the Fisher test in case of mutual exclusive categories of the dependent and independent variable without adjustment. Regression coefficients with respective confidence intervals are listed in Supplementary Table S2; *significant on an α-level of 5%; *NRS* Numeric rating scale.

Within group comparisons showed that under the thermal grill condition both, fibromyalgia patients and healthy controls, rated the warm/heat sensation (Fig. 3b) as well as unpleasantness significantly higher (Fig. 3c) and reported burning (Fig. 3e) significantly more frequently than during contact with the 20 °C- or 40 °C-thermal plates (*p* ≤ 0.02). Compared to the 20 °C-evoked cold sensation, the thermal grill-evoked cold sensation was not significantly different in healthy controls (*p* = 0.255) but significantly stronger in fibromyalgia patients (*p* = 0.011; Fig. 3a). In addition, in fibromyalgia patients but not in healthy controls, levels of pain were significantly higher during the thermal grill condition compared to the uniform 20 °C- or 40 °C-condition (*p* < 0.001 and *p* = 0.068, respectively; Fig. 3d). In cases with 20 °C- and/or 40 °C-evoked pain one cannot speak of a “pure” TGI. Therefore, these cases are marked in Fig. 4 which illustrates the main finding of the association analyses described in the following.

### Associations of the thermal grill illusion with sensory thresholds

#### Consistent positive associations between temporal summation (TS) and thermal grill-evoked sensations in fibromyalgia patients

Among fibromyalgia patients, TS was positively associated with the intensity of the thermal grill-evoked warm/heat sensation and unpleasantness as well as with the occurrence of thermal grill-evoked pain. For a one unit increase of the logarithmized WUR (WUR_log_), which corresponds to a WUR difference of 1 versus 10, the thermal grill-evoked warm/heat sensation increased by 20.78 points on the NRS (95%-CI [1.57; 39.99], *p* = 0.034) and unpleasantness by 29.42 NRS points (95%-CI [0.12; 58.72], *p* = 0.049). The odds ratio (OR) for experiencing thermal grill-evoked pain was 57.31 (95%-CI [1.86; 1764.41], *p* = 0.021) for a one unit increase of the WUR_log_. These associations remained significant after adjustment for covariates. TS in fibromyalgia patients was neither significantly associated with the intensity of the cold sensation nor with the pain intensity among those with pain under the thermal grill condition. (Fig. 4, Supplementary Table S1).

Among healthy controls, who exhibited overall lower and less extreme TS, stronger thermal grill-evoked unpleasantness was significantly associated with lower TS (β [95%-CI] for WUR_log_ −43.31 [−78.59;  − 8.02], *p* = 0.016; Supplementary Table S2).

#### Little association between thermal detection or pain thresholds and the thermal grill illusion

In fibromyalgia patients, a loss in the warm detection threshold (WDT) was significantly associated with higher pain ratings among those with pain under the thermal gill condition (β [95%-CI] for WDT_log_ 55.75 [6.70; 104.79], *p* = 0.026). However, this association did not remain significant after adjustment for covariates (β [95%-CI] 50.53 [−0.63; 101.68], *p* = 0.053, Supplementary Table S1). In contrast, in healthy controls who showed a lower and narrower WDT value range, a higher WDT was significantly associated with a less intense thermal grill-evoked warm/heat sensation (β [95%-CI] for WDT_log_−44.01 [−79.93; −8.09], *p* = 0.016; Supplementary Table S2). No further significant associations between thermal detection thresholds (CDT, WDT and TSL) and thermal grill-evoked sensations were observed.

In fibromyalgia patients, a cold pain threshold (CPT) at higher temperatures was significantly related to a higher intensity of the thermal grill-evoked cold sensation (β [95%-CI] 0.81 [0.14; 1.47], *p* = 0.017; Supplementary Table S1). This association remained significant in the adjusted analysis. In addition, a CPT at higher temperatures was significantly related to the chance of experiencing 20 °C-evoked unpleasantness (OR [95%-CI] 1.10 [1.01; 1.21], *p* = 0.030; Supplementary Table S4). In healthy controls, the CPT was neither significantly associated with thermal grill-evoked sensations nor with 20 °C-evoked sensations (Supplementary Table S2).

No significant relationship was found between the heat pain threshold (HPT) and any of the thermal grill-evoked sensations; neither in fibromyalgia patients nor in healthy controls (Supplementary Tables S1 and S2). A HPT at lower temperatures was significantly associated with the intensity of the 40 °C-evoked warm sensation in both, fibromyalgia patients (β [95%-CI]  − 1.11 [−2.01;  − 0.21], *p* = 0.016) and healthy controls (β [95%-CI]  − 1.66 [−3.02;  − 0.31], *p* = 0.016; Supplementary Table S4). No other significant relationships were found between the HPT and 40 °C-evoked sensations.

#### Little association between mechanical detection and pain thresholds and the thermal grill illusion

In fibromyalgia patients, a loss of the vibration detection evaluated by the method of Limits (VDT_Limit_) was positively related to a higher thermal grill-evoked warm/heat sensation (β [95%-CI] −17.25 [−28.81;  − 5.68], *p* = 0.003). In addition, a loss of the vibration detection evaluated by the method of Levels (VDT_Levels_) was positively associated with the thermal grill-evoked pain sensation among those with pain (β [95%-CI] 18.48 [0.11; 36.85], *p* = 0.049), albeit this association was not significant after adjustment for covariates. After adjustment for covariates, a MDT at higher forces was significantly associated with a higher thermal grill-evoked warm/heat sensation (β for MDT_log_ [95%-CI]  − 14.98 [−29.67;  − 0.29], *p* = 0.046]. The mechanical pain threshold (MPT), mechanical pain intensity (MPS), dynamic mechanical allodynia (DMA) and the pressure pain threshold (PPT) were not significantly associated with any thermal grill-evoked sensation in fibromyalgia patients (Supplementary Table S1).

In healthy controls, a higher PPT was significantly related to a lower probability of thermal grill-evoked burning (OR 95%-CI for PPT_log_ 6.03*10^–8^ [5.62*10^–15^; 0.65], *p* = 0.044; Supplementary Table S2).

### Associations of the thermal grill illusion with thermal perception

#### Consistent associations between cold (20 °C)-evoked sensations and thermal grill-evoked sensations

Associations between the cold (20 °C)-evoked sensations and thermal grill-evoked sensations are depicted in Fig. 4 and Supplementary Table S3. In fibromyalgia patients, the intensity of the cold (20 °C)-evoked cold sensation was positively associated with the intensities of the thermal grill-evoked sensations: cold sensation (β [95%-CI] 0.88 [0.62; 1.15], *p* < 0.001), warm/heat sensation (β [95%-CI] 0.65 [0.36; 0.93], *p* < 0.001), unpleasantness (β [95%-CI] 1.11 [0.71.; 1,51], *p* < 0.001) and pain among those with pain (β [95%-CI] 0.75 [0.25; 1.24], *p* = 0.003). The associations remained significant after adjustment for covariates. In contrast, the 20 °C-evoked cold sensation was not associated with the occurrence of thermal grill-evoked pain or burning.

Patients who reported 20 °C-evoked unpleasantness (n = 26) experienced the thermal grill-evoked sensations significantly more intense than those without 20 °C-evoked unpleasantness (Fig. 4, Supplementary Table S3). On average the experience of 20 °C-evoked unpleasantness was significantly associated with a 19.45 (95%-CI [9.03; 29.87], *p* < 0.001) point higher intensity of the cold sensation (median NRS [IQR] 44.1 [36.7; 60.0] vs 21.7 [17.9; 36.3]), a 11.93 (95%-CI [1.52; 22.34], *p* = 0.025) point higher intensity of the warm/heat sensation (median NRS [IQR] 58.3 [45.4; 68.3] vs. 44.2 [29.2; 63.8]) and a 25.32 (95%-CI [10.54; 40.10], *p* = 0.001) point higher intensity of unpleasantness (median NRS [IQR] 51.7 [27.9; 67.5] vs. 16.7 [6.7; 38.3]) under the thermal grill condition. Additionally, 20 °C-evoked unpleasantness was significantly associated with a greater chance of pain (20 out of 26 vs 5 out of 14, OR [95%-CI] 6.00 [1.44; 24.92], *p* = 0.014) and burning (21 out of 26 vs 4 out of 14, OR [95%-CI] 10.50 [2.31; 47.78], *p* = 0.002) under the thermal grill condition. All these associations remained significant after adjustment for covariates despite the association between 20 °C-evoked unpleasantness and the thermal grill-evoked warm/heat sensation. Five of the patients reporting 20 °C-evoked unpleasantness also experienced thermal grill-evoked burning. In these patients, the intensity of such unpleasantness was significantly related to thermal grill-evoked burning (β [95%-CI] 0.89 [0.98; 0.80], *p* = 0.020).

All patients feeling pain during contact with the 20 °C plate (n = 10) also reported thermal grill-evoked pain, while this was only the case for half of the patients without 20 °C-evoked pain (15 out of 30; *p* = 0.006). In addition, all patients with a burning sensation during contact with the 20 °C plate (n = 8) also reported thermal grill-evoked burning. Among patients without 20 °C-evoked burning, 53% reported thermal grill-evoked burning (15 out of 32, *p* = 0.016). Patients with 20 °C-evoked burning also reported a more intense thermal grill-evoked warm/heat sensation (β [95%-CI] 15.42 [3.14.; 27.69], *p* = 0.014, median NRS [IQR] 61.7 [56.7; 79.2] vs. 48.3 [38.3; 63.3]), albeit this association was not confirmed in the adjusted analyses. No other associations between 20 °C-evoked pain or burning and thermal grill-evoked sensations were observed.

Furthermore, combined regression analyses revealed that associations of thermal grill-evoked sensations with the 20 °C-evoked cold sensation and unpleasantness were statistically independent from its associations with TS (Supplementary Table S5).

In healthy controls, the intensity of the 20 °C-evoked cold sensation was also positively associated with the intensity of the thermal grill-evoked cold sensation (β [95%-CI] 0.82. [0.37; 1.27], *p* < 0.001) and warm/heat sensation (β [95%-CI] 0.55 [0.12; 0.99], *p* = 0.013). The 20 °C-evoked cold sensation was not significantly associated with the thermal grill-evoked experiences of unpleasantness, pain or burning. (Supplementary Table S2).

#### Little association between warm (40 °C)-evoked sensations and thermal grill-evoked sensations

A higher rating of the intensity of the 40 °C-evoked warm sensation was significantly related to a higher intensity of the thermal grill-evoked cold sensation in fibromyalgia patients (β [95%-CI] 0.68 [0.26; 1.10], *p* = 0.002; significant also after adjustment for covariates). The intensity of 40 °C-evoked unpleasantness, experienced by 30% of the fibromyalgia patients (n = 12), was significantly related to the intensity of the thermal grill-evoked cold sensation (β [95%-CI] 0.73 [0.01; 1.44], *p* = 0.047), unpleasantness (β [95%-CI] 1.29 [0.48; 2.10], *p* = 0.002) and pain (β [95%-CI] 1.19 [0.57; 1.81], *p* < 0.001). The three patients with 40 °C-evoked pain also experienced pain during the thermal grill condition. A 40 °C-evoked burning sensation was experienced by 28% of the patients (n = 11). Among those, 91% (10 out of 11) also reported burning under the thermal grill condition. Approximately half of the patients without 40 °C-evoked burning (15 out of 29) experienced thermal grill-evoked burning (OR [95%-CI] 9.33 [1.05; 82.64], *p* = 0.045), albeit this association was not significant after adjustment for covariates. (Supplementary Table S3).

In healthy controls, the 40 °C-evoked warm sensation was positively related to the thermal grill-evoked cold (β [95%-CI] 0.86 [0.42; 1.29], *p* < 0.001) and warm/heat sensation (β [95%-CI] 0.64 [0.23; 1.06], *p* = 0.002); Supplementary Tables S2).

## Discussion

This is the first study exploring associations between sensory profiles evaluated by standardized QST and the TGI in fibromyalgia patients. The comparison with healthy controls illustrates an enhancement of thermal grill-evoked sensations, stronger and more aversive thermal perception and distinct characteristics of the sensory profile, namely a loss in warm, mechanical and vibration detection as well as a gain in cold and heat pain thresholds and increased TS. The identified relationships between TGI augmentation and altered sensory processing in fibromyalgia patients in comparison to healthy controls contribute to a deeper understanding of the TGI mechanisms. Among the 13 QST parameters measured in the present study, TS was most closely related to the TGI, in particular with pain and unpleasantness. Additionally, thermal grill-evoked sensations were consistently associated with intense and aversive cold (20 °C)-evoked sensations but less with warm (40 °C)-evoked sensations and not with thermal detection and pain thresholds.

### Facilitation of the pain pathway augments the thermal grill illusion

The fact that high TS in fibromyalgia patients was associated with an augmented TGI, i.e. the thermal grill-evoked warm/heat sensation, unpleasantness as well as the occurrence of pain, supports the existing evidence of an activation of the pain pathway during the TGI^13,14,33^ and is also suggested by recent work of another research group^34^. Pin-prick induced TS is interpreted as a marker for central, in particular spinal, synaptic facilitation within the pain pathway^35^. As in previous studies^28,36^, TS was increased in fibromyalgia patients compared to healthy controls. Augmentation of the TGI by spinal fascilitatory pain processing seems to be specific for patients suffering from fibromyalgia, since in healthy controls TS (in a lower and narrower range) was negatively associated with thermal grill-evoked unpleasantness. It seems possible that in the healthy nervous system pain fascilitation and endogenous pain control mechanisms are balanced, while in fibromyalgia patients this balance might be disrupted. Indeed, high TS in combination with low conditioned pain modulation (CPM), as a measure for descending pain control mechanisms, has been observed in fibromyalgia patients^28,37^. In turn, high TS in healthy controls seems to be at least in part counteracted by more functional CPM^38^.

The associations of thermal grill-evoked sensations with high TS in fibromyalgia patients were statistically independent of the associations between thermal grill-evoked sensations and 20 °C-evoked sensations (see below). This suggests that pain facilitation and alterations in the interplay between the thermoreceptive and the nociceptive system are two separate mechanisms, both contributing to an augmented TGI.

### Strong and aversive cold processing enhances the thermal grill illusion

The close relationship between thermal grill-evoked sensations and cold (20 °C)-evoked sensations supports the previous notion that the cold input is a major determinant of the TGI. In healthy volunteers, the TGI was found to be decreased after cold but not after warm adaptation^39^, and it was enhanced by TRPM8 and TRPA1 agonists^17^, whereby TRPM8 and TRPV1 receptors characterize the vast majority of cold sensors^40^. The findings have been interpreted in the light of Craig’s and Bushnell`s hypothesis that the TGI results from an unmasking of cold-evoked C-fiber input to the pain pathway which is inhibited through Aδ-fiber dependent central mechanisms during innocuous cold stimulation^13,14^. Accordingly, the previously observed correlation of a stronger TGI with a CPT at higher temperatures in healthy volunteers^17–20^ and a decreased TGI together with a CPT at lower temperatures in patients with psychiatric disorders^23–25^ is thought to reflect that the disinhibition and the sensitivity of C-fibers plays a role in both, the TGI and the elicitation of cold pain. Analogously, cold hyperalgesia in neuropathic pain is thought to be caused by a selective loss of Aδ-fiber function and C-fiber sensitization^41,42^. However, most likely due to the activation of cold nociceptors during cold pain perception but not during the TGI, and a different impact of affective pain processing, correlations between the CPT and the TGI were found to be sometimes weak (r between 0.2 and 0.3)^19,20^ or fully absent as in healthy females in a previous^18^ and the present study.

Also in fibromyalgia patients included in the present study, the CPT was only associated with the thermal grill-evoked cold sensation, despite its association with the interpretation of the 20 °C-stimulus as unpleasant in fibromyalgia patients. It seems unlikely that the enhanced TGI in the fibromyalgia group was caused by impaired inhibitory control through a loss of Aδ-fiber function, since the CDT (that is deemed to indicate Aδ-fiber function) was neither different from the healthy control group nor was it related to any of the thermal grill-evoked sensations. Therefore, the results of our study suggest a minor role of cold sensitive peripheral nerve fiber function for the augmented TGI in fibromyalgia patients. Even in those patients who experienced 20 °C- and 40 °C-evoked pain and consequently also thermal grill (20 °C/40 °C)-evoked pain, the intensity of pain during the thermal grill condition was higher than the pain intensity during the uniform thermal stimuli and not associated with it. Strictly speaking, in those patients with 20 °C- and/or 40 °C-evoked pain, one cannot speak of a classical TGI during the thermal grill condition, which by definition occurs with the application of innocuous thermal stimuli. However, presenting these cases separately in Fig. 4 shows that with the exception of pain, these patients exhibited the full spectrum of thermal grill-evoked sensations, as did their peers with no 20 °C- or 40 °C-evoked pain.

In view of the supposedly marginal role of the function of cold sensitive peripheral nerve fibers, a predominant role of altered central cold processing for the augmented thermal grill-evoked sensation seems possible. The enhanced 20 °C-evoked cold sensation and the enhanced 20 °C-evoked unpleasantness in fibromyalgia patients were strongly associated with a more intense cold and warm sensation, stronger pain and unpleasantness and a higher chance of burning under the thermal grill condition. These observations are particularly striking as they contrast the comparatively little associations between the thermal grill-evoked sensations and the 40 °C-evoked sensations. It seems that the overall aversive interpretation of cold in fibromyalgia patients predicts the recruitment of pain circuits during the TGI. This appears plausible given the relationship between aversive cognitions, such as catastrophizing, and an enhanced attentional and affective pain processing in fibromyalgia^43^ and an augmentation of the TGI by negative affect and pain expectations^44–46^. This coincides with the observation that in healthy controls, who did not experience contact with the 20 °C cold-plate as aversive, i.e. unpleasant, painful or burning, the 20 °C-evoked cold sensation was only associated with the cold and warm/heat sensation but not with sensations indicating a pain pathway activation during the thermal grill condition. Negative conditioning against thermal stimuli might contribute to an impaired general endogenous pain control, e.g. the descending inhibitory pathways originating in the periaquaeductal grey that functions independently of pain inhibition through cold processing centers^47^. Indeed, endogenous pain control quantified by CPM has been shown to reduce the TGI^48^. CPM is commonly impaired in fibromyalgia patients^28^ and diminished in healthy volunteers by pain catastrophizing^49,50^. Future research should explore the relationship between the conditioned pain modulation and the TGI in fibromyalgia.

### Warm processing plays a minor role in the thermal grill illusion

In contrast to TS and 20 °C-evoked sensations, warm (40 °C)-evoked sensations were less consistently related to the TGI. The 40 °C-evoked warm sensation was only associated with the thermal grill-evoked cold sensation in fibromyalgia patients and with the thermal grill-evoked cold and warm/heat sensation in healthy controls, but not with the specific TGI sensations unpleasantness, pain and burning. The relationships between the intensities of 40 °C-evoked unpleasantness and thermal grill-evoked unpleasantness and pain as well as between 40 °C- and thermal grill-evoked burning supports the statement made above that averseness against thermal stimuli might contribute to pain pathway activation during the TGI. Thus, warm processing seems to be less decisive for the intensity of thermal grill-evoked sensations, although, the warm input is certainly necessary to elicit the TGI in the first place^21^. The intense but not necessarily painful warm/heat sensation during the TGI cannot be fully explained by the disinhibition theory. Our results support the previous notion that heat nociceptors are not at play here^17,18,39^. The HPT was not associated with the TGI, neither in fibromyalgia patients nor in healthy controls, despite its association with the 40 °C-evoked warm sensation. Furthermore, it seems unlikely that hyperexcitability of warm sensors is a main driver for the augmented TGI in fibromyalgia patients, as the WDT was solely associated with the intensity of pain among those with pain (see next paragraph). Recent findings in mice indicate that, besides activation of warm-receptors (polymodal C-fibers), warm perception also depends on the deactivation of polymodal TRPM-8 positive C-fibers responding to skin temperature changes^51^. Thus, adjacent cool and warm elements of the thermal grill-plate might mimic skin temperature changes eliciting the intense warm sensation. Such integration of multichannel input would be in accordance with the population coding theory proposed for the TGI^6^.

### Role of loss in peripheral nerve fiber function for the TGI

In the present study, fibromyalgia patients showed an increased WDT compared to healthy controls with at least 25% exhibiting values that indicate a substantial loss of small-fiber function. This has been found in previous studies using QST and skin biopsy analyses^52,53^. In addition, the patient population in our study also showed impaired detection of punctate mechanical stimuli (MDT) and vibration (VDT) which may be related to a large fiber-pathology also previously observed in fibromyalgia^52,54^. Impaired warm, mechanical and vibration detection were each associated with only one of the five thermal grill-evoked sensations determined in this study. This might suggest that peripheral nerve fiber affection in fibromyalgia patients is not a main driver of the augmented TGI in this population. Furthermore as expected, small fiber function in healthy women (WDT in the healthy range) covaried positively with the warm sensation under the thermal grill condition.

### Limitations

Estimates of the association measures are based on an exploratory analysis that comes with the downsides of multiple testing. Thus, part of the detected associations could be based on chance and require replication in future studies. Albeit, the clear patterns of associations between the thermal grill-evoked sensations and high TS as well as cold-evoked sensations clearly point to a relevant role of the mechanisms discussed above. These associations remained unimpacted by adjustment for several covariates, including the three DASS subscores which demonstrate the naturally higher levels of depression, anxiety and stress in fibromyalgia patients compared to healthy controls. In addition, QST as well as the assessments of cold-, warm- and thermal grill-evoked sensations represent psychophysiological tests that are prone to various influences. For example, sensations evoked by sensory assessments might have been interpreted differently by fibromyalgia patients and healthy controls; a phenomenon also described as differences in decision criterion^55^. This was reduced by the use of standardized instructions, i.e. the QST-protocol developed by the DFNS, for all participants in our study (see supplementary Appendix 1 of^56^). Bias during sensory assessments was further minimized by only recruiting participants naïve to the TGI phenomenon and by having all measurements performed in the same stable-temperature room by female examiners. The association analyses in fibromyalgia patients and healthy controls cannot be directly compared due to the different sample sizes which affect the power of the association analyses. Direct comparisons of associations of measured outcomes between fibromyalgia patients and healthy controls are generally hardly tenable due to the fact that value ranges differ both in location and divergence. The healthy control group was primarily implemented in this study to quantify how sensory profiles and thermal processing differ in fibromyalgia patients from a healthy population.

## Conclusion

The obtained results suggest that the cold input is the main determinant of the TGI, and that central facilitation further augments the activation of the pain pathway during the TGI. Enhanced temporal summation and increased cold processing, in particular an aversive interpretation of cold were identified as independent factors augmenting the TGI in fibromyalgia patients. From a clinical perspective, this suggests that in addition to central pain facilitation and reduced PAG mediated endogenous pain control, strong aversive cold processing contributes to central disinhibition and thereby to excessive activation of the pain pathway in fibromyalgia. Consequently, deconditioning against cold might be another important element of multimodal treatment concepts for fibromyalgia that are already recommended as the basis for non-pharmacological therapies^30,57^. Given these considerations, the TGI might play a potential role in assisting the design and evaluation of mechanism-based treatments.

## Methods

### Study design and setting

In 40 female patients diagnosed with fibromyalgia and 20 healthy women, thermal grill-evoked sensations as well as sensations induced by contact with a cold (20 °C) or a warm (40 °C) thermal plate were assessed on the palm of the dominant hand. In addition, sensory profiles at the thenar eminence of the same hand were evaluated by standardized QST. Assessments of thermal grill-evoked sensations and sensory profiles were performed at an interval of two to maximum four days in randomized order to minimize effects of serial testing. Patients were included between October 2018 and October 2019 and healthy controls between August 2021 and January 2022. Both, the thermal grill experiments and QST were carried out by the same examiners (AB, PB, HA) at the Multidisciplinary Pain Center, Department of Anaesthesiology, University Hospital LMU Munich, Germany.

### Patients

Patients included in this study had to be female, between 18 and 60 years old and diagnosed with fibromyalgia according to the criteria defined in the German medical guideline including the criteria of the American College of Rheumatology (ACR 2010/2016 criteria)^30,31^. Diagnosis had to be established or reconfirmed by physicians of one of the recruiting study centers. Male gender and severe or moderate depression (clinical diagnosis of depression or depression subscore of the depression, anxiety and stress scales (DASS) > 13 which indicates moderate or severe depression) were specific exclusion criteria. Both characteristics are known to impact sensory profiles and/or thermal grill-evoked sensations^18,24,58^ with less pronounced thermal grill-evoked sensations associated with male gender^18^ and depression^24,46^ and lower pain thresholds in men^56^. Mild depressive symptoms, however, did not lead to exclusion as they are part of the fibromyalgia syndrome^31^. Patients with malignant, rheumatoid or chronic inflammatory diseases, severe mental diseases, mono- or polyneuropathies not related to fibromyalgia and patients taking anticonvulsants, antidepressants or opioids were excluded. Patients were asked not to take any analgesic medication within the 24 h prior to the study visits.

The healthy control group was recruited after measurements in the patient group were completed. Healthy women were matched for age with the included patient population. Healthy volunteers were asked whether they had been diagnosed with a malignant, rheumatoid or chronic inflammatory disease, whether they suffered from other chronic complaints or psychological symptoms, whether they had received a psychological or psychiatric diagnosis and whether they took medication regularly. If this was confirmed, volunteers were not included. In addition, volunteers with DASS depression subscore > 9 (cut-off for a normal values, no depressive symptoms) were not included in the study.

Further inclusion criteria for both, fibromyalgia patients and healthy controls, were good command of the German language, voluntary participation and written informed consent. Exclusion criteria applying to both groups were allergies to cupper or nickel, acute illness or trauma and pregnancy.

Patient recruitment was performed through announcements on information boards, direct patient contact via telephone and at information events in the Multidisciplinary Pain Center, Department of Anaesthesiology and the Department of Orthopaedics and Trauma Surgery, Musculoskeletal University Center Munich (MUM), University Hospital LMU Munich, Germany. Healthy controls were recruited through information sheets that were available in the staff facilities of the University hospital LMU Munich and of the Biomedical Center Munich, University of Munich. Additionally, announcements for fibromyalgia patients and healthy controls were sent by e-mail to voluntary recipients via the official information service of the LMU (“LMU Infodienst”).

### Thermal grill illusion (TGI)

The thermal grill experiments were performed by trained examiners (AB and PB). All participants were naïve about the TGI. They were informed about the rating procedure and assured that all stimulation parameters were harmless. For stimulation, the participants placed the palmar surface of the dominant hand on a thermal plate that was fixed to a table. The set-up of the thermal grill device has been described elsewhere^17,18^. Briefly, the thermal plate consists of 15 nickel bars (tubes) that are perfused with warm or cold water. The applied temperatures were 20 °C, 40 °C and 20 °C alternating with 40 °C (thermal grill condition). A second thermal plate with all bars held at 32 °C was used to establish a baseline temperature of the skin immediately prior to each thermal stimulation trial. To examine sensations associated with thermal stimulations, the hand was first placed on the 32 °C reference plate for 20 s. The hand was then exposed to a uniform 20 °C stimulus for 20 s and the participant was asked to specify the evoked perceptions by using the descriptors "warm/heat, cold, unpleasantness, pain and burning". Then, the participant was presented with numeric rating scales (NRS) to indicate the intensity of a) the cold sensation, b) the warm sensation, c) the feeling of unpleasantness and d) of pain. The NRS ranged from 0 to 100 with increments of 10 resulting in the following possible ratings: 0, 10, 20, 30, 40, 50, 60, 70, 80, 90, 100. Zero represented a neutral temperature sensation, no unpleasantness or no pain, and 100 represented worst cold, worst warm/hot, worst unpleasantness or pain that could be imagined. This procedure was repeated with the application of a uniform 40 °C stimulus followed by the interleaved 20/40 °C (grill) stimulus, each intermitted by placing the hand on the control plate (32 °C) for 20 s. The test series with the three stimuli (uniform 20 °C, uniform 40 °C and interleaved 20/40 °C) was presented three times with a minimum inter-stimulus interval of five minutes.

### Quantitative sensory testing

Sensory profiles were assessed by QST according to the DFNS standardized protocol by certified examiners (AB, HA and PB). Each participant was tested on the palm of the dominant hand. The DFNS QST protocol has been published in detail^42^ and includes determination of 13 sensory parameters: the cold and warm detection threshold (CDT, WDT), the thermal sensory limen (TSL), paradoxical heat sensations (PHS), the cold and heat pain threshold (CPT, HPT), the mechanical detection and pain threshold (MDT, MPT), mechanical pain sensitivity (MPS), dynamic mechanical allodynia (DMA), the wind-up ratio (WUR) as a measure for temporal summation (TS) of pain, the vibration detection threshold (VDT) and the pressure pain threshold (PPT). Thermal stimuli were applied by a Peltier-based computerized thermal stimulator (TSA II, Medoc, Ramat Ishai, Israel, 30 × 30 mm contact probe). For mechanical testing von Frey filaments (0.25 to 512 mN; MARSTOCK nervtest, Marburg, Germany), blunt pinpricks (8 to 512 mN; Department of Physiology and Pathophysiology, Mainz, Germany) and a hand held pressure algometer (2 to 10 kg; 1cm^2^ contact probe; FDK20, Wagner Instruments, Greenwich, CT) were used. The WUR was calculated as the ratio of the pain intensities evoked by 1 and 10 pin-prick stimuli of 256 mN (if not tolerated 128 mN). Vibration detection was evaluated by the method of Limits by using the Rydel-Seiffer tuning fork (64 Hz) with an amplitude scale from zero to eight. In addition, the VDT was also tested by the method of Levels using the Vibration Sensory Analyzer-3000 (VSA-3000, Medoc, Ramat Ishai, Israel).

### Randomization of testing sequence

An open source software tool (https://www.randomizer.org/) was used to assign group allocation to each participant identification number. Allocation concealment was achieved by using sealed opaque envelopes that were labeled with the patient or healthy volunteer identification number and contained a letter with the group allocation. Envelopes were prepared by an independent researcher not involved in the experiments (KH) and were kept in the secretary's office of the study center. Subsequent to inclusion, the participant was assigned a consecutive patient or healthy volunteer identification number and the examiner opened the respective randomization envelope.

### Participant characteristics

During screening, paraticipants were asked to fill out the depression, anxiety and stress scales (DASS)^59^, as a depression subscore below 13 or below 9 was mandatory for inclusion of fibromyalgia patients and healthy volunteers, respectively (see above). The DASS is a 21-item self-reported measure with seven items each on symptoms of depression, anxiety and stress which are coded from zero to three. The three subscores are calculated by building the sum of the respective items. In case of inclusion, the DASS questionnaire was kept and the age of the participant was documented on the case report form. For each fibromyalgia patient pain duration, current medication, stage of chronicity according to the Mainz Pain Staging System (MPSS)^60^ as well as current medication were extracted from medical records. At the first study visit, patients were asked to verify and complete this information as appropriate and to indicate their pain intensity over the last week on a NRS (0–10).

### Biometry

The sample size of the patient group was estimated to detect a difference of one standard deviation between subgroups representing 33% and 66% of the whole sample on an alpha level of 5% with a power of 80%. The sample size of the healthy control group added a posteriori was based on the following consideration: Based on the previously established values for 20 °C-, 40 °C- and thermal grill-evoked sensations as well as thermal detection and pain thresholds at the palm of the hand in healthy subjects, one standardized mean difference or more can be considered clinically relevant^17,18^. Given the group size of fibromyalgia patients (n = 40) and assuming an alpha error of 5% and a power of 80%, it was calculated that a healthy control group of size 13 was sufficient to detect an SMD of 1 by the Mann–Whitney U test. To account for a potential loss of power due to the skewed distributions in the fibromyalgia patient group and to reflect the age distribution of the patient population in the healthy control group, we included 20 age-matched healthy women. Data analysis was carried out by using the statistical software SPSS (IBM SPSS Statistics 25.0). According to the QST-analysis protocol of the DFNS, the QST parameters except for the CPT, HPT, PHS and VDT were log-transformed in order to approximate normal distribution. Since the distribution of several continuous variables deviated from normal (especially among fibromyalgia patients), the data are presented as medians with interquartile ranges. Regarding the sensations evoked by 20 °C, 40 °C and the thermal grill stimulus (measured on a continuous scale), which did not occur in a substantial proportion of cases (NRS = 0 in 20% or more), separate descriptives were provided for the subgroup reporting these sensations. Minimum and maximum values are reported only when continuous sensory parameters occurred in less than five cases. Categorical variables are presented as absolute and relative frequencies. Group comparisons were performed by the Mann–Whitney-U test for continuous data and by the Fisher Test for dichotomous data. Within group comparisons were performed by the Wilcoxon rank-sum test or the McNemar test, respectively.

Associations of thermal grill-evoked sensations with 20 °C- and 40 °C-evoked sensations as well as with QST parameters were evaluated by generalized linear models (GLM) employing maximum likelihood estimation. GLM were fitted assuming a normal distribution and an identity link function for continuous data and a binomial distribution with a logit link function for dichotomous data. Separate analyses were run for each thermal grill-evoked sensation as the dependent variable with one predictor variable (each QST parameter as well as each 20 °C- and each 40 °C-evoked sensation) at a time. Two separate association analyses were performed for continuous variables quantifying sensations evoked by 20 °C, 40 °C and the thermal grill-stimulus which were not perceived in a substantial proportion of cases. First, the sensations were treated as binary variables (yes means NRS > 0, no means NRS = 0), and second, they were treated as continuous variables in subgroup analyses among patients experiencing the respective sensation. PHS as well as well as a warm sensation during contact with the cold plate (20 °C) and a cold or pain sensation during contact with the warm plate (40 °C) occurred too infrequent to be assessed for an association with thermal grill-evoked sensations. Dynamic mechanical allodynia (DMA) was analyzed as a dichotomous variable as pain intensity evoked by light stroking was very low (< 2 on NRS 0–100) in all patients showing DMA.

Crude and covariate adjusted regression coefficients were estimated, except for regression of subgroup data and data obtained in healthy controls in which covariate adjustment was not meaningful due to the small case numbers. Covariates for adjusted analyses included age, DASS depression subscore, DASS anxiety-subscore, DASS stress subscore and pain intensity in the last week before inclusion in the study. Analyses were not adjusted for stage of chronicity and pain duration in order to avoid over adjustment^61^, since pain duration can be interpreted as an ascending proxy and stage of chronicity as a descending proxy of the independent variables representing neuroplastic changes that develop over time and contribute to pain chronification. If both, the dependent and independent variable, were dichotomous with mutually exclusive categories, the Fisher test was applied for unadjusted analyses, and adjusted analyses were omitted because GLM are not applicable in this case. *P*-values are reported without adjustment for multiple testing according to the explorative design of the study.

### Ethical considerations

The study was conducted in accordance with the Declaration of Helsinki^62^ and approved by the Ethics Committee of the Medical Faculty of the Ludwig Maximilian University (LMU), Munich, Germany. Participants were informed in oral and written form, participated voluntarily and provided written informed consent. Participants could withdraw from the study at any time and received a financial remuneration after completing the second study visit. Data were handled pseudonymously in accordance with the German data-protection act and are kept in the research facilities of the Multidisciplinary Pain Center, Department of Anaesthesiology, LMU University Hospital, LMU Munich, Munich Germany. Data collected from participants who were screened but not included in the study were deleted.

### Supplementary Information

Supplementary Tables.

## Data Availability

Data of this study and the SPSS syntax used for the analyses are available via the digital object identifier 10.6084/m9.figshare.13667354.

## References

[1] Alrutz S (1898). On the temperature senses: II. The sensation 'hot'. Mind.

[2] Adam F, Alfonsi P, Kern D, Bouhassira D (2014). Relationships between the paradoxical painful and nonpainful sensations induced by a thermal grill. Pain.

[3] Leung AY, Wallace MS, Schulteis G, Yaksh TL (2005). Qualitative and quantitative characterization of the thermal grill. Pain.

[4] Hunter J, Dranga R, van Wyk M, Dostrovsky JO (2015). Unique influence of stimulus duration and stimulation site (glabrous vs. hairy skin) on the thermal grill-induced percept. Eur. J. Pain..

[5] Bach P, Becker S, Kleinböhl D, Hölzl R (2011). The thermal grill illusion and what is painful about it. Neurosci. Lett..

[6] Fardo F, Beck B, Allen M, Finnerup NB (2020). Beyond labeled lines: A population coding account of the thermal grill illusion. Neurosci. Biobehav. Rev..

[7] Ma Q (2010). Labeled lines meet and talk: population coding of somatic sensations. J. Clin. Invest..

[8] Forstenpointner J, Berry D, Baron R, Borsook D (2020). The cornucopia of central disinhibition pain-An evaluation of past and novel concepts. Neurobiol. Dis..

[9] Mackenzie RA, Burke D, Skuse NF, Lethlean AK (1975). Fibre function and perception during cutaneous nerve block. J. Neurol. Neurosurg. Psychiatry..

[10] Wahren LK, Torebjork E, Jorum E (1989). Central suppression of cold-induced C fibre pain by myelinated fibre input. Pain.

[11] Wasner G, Schattschneider J, Binder A, Baron R (2004). Topical menthol–a human model for cold pain by activation and sensitization of C nociceptors. Brain: J. Neurol..

[12] Yarnitsky D, Ochoa JL (1990). Release of cold-induced burning pain by block of cold-specific afferent input. Brain : J. Neurol..

[13] Craig AD, Bushnell MC (1994). The thermal grill illusion: Unmasking the burn of cold pain. Science.

[14] Craig AD, Reiman EM, Evans A, Bushnell MC (1996). Functional imaging of an illusion of pain. Nature.

[15] Hensel H, Boman KK (1960). Afferent impulses in cutaneous sensory nerves in human subjects. J. Neurophysiol..

[16] Campero M, Baumann TK, Bostock H, Ochoa JL (2009). Human cutaneous C fibres activated by cooling, heating and menthol. J. Physiol..

[17] Averbeck B, Rucker F, Laubender RP, Carr RW (2013). Thermal grill-evoked sensations of heat correlate with cold pain threshold and are enhanced by menthol and cinnamaldehyde. Eur. J. Pain..

[18] Averbeck B, Seitz L, Kolb FP, Kutz DF (2017). Sex differences in thermal detection and thermal pain threshold and the thermal grill illusion: A psychophysical study in young volunteers. Biol. Sex. Differ..

[19] Lindstedt F, Lonsdorf TB, Schalling M, Kosek E, Ingvar M (2011). Perception of thermal pain and the thermal grill illusion is associated with polymorphisms in the serotonin transporter gene. PLoS ONE.

[20] Schaldemose EL, Horjales-Araujo E, Svensson P, Finnerup NB (2015). Altered thermal grill response and paradoxical heat sensations after topical capsaicin application. Pain.

[21] Bouhassira D, Kern D, Rouaud J, Pelle-Lancien E, Morain F (2005). Investigation of the paradoxical painful sensation ('illusion of pain') produced by a thermal grill. Pain.

[22] Green BG (2002). Synthetic heat at mild temperatures. Somatosens. Mot. Res..

[23] Bekrater-Bodmann R (2015). Deficits in pain perception in borderline personality disorder: Results from the thermal grill illusion. Pain.

[24] Boettger MK, Grossmann D, Bar KJ (2013). Thresholds and perception of cold pain, heat pain, and the thermal grill illusion in patients with major depressive disorder. Psychosom. Med..

[25] Boettger MK, Grossmann D, Bär KJ (2013). Increased cold and heat pain thresholds influence the thermal grill illusion in schizophrenia. Eur. J. Pain.

[26] Sumracki NM, Buisman-Pijlman FT, Hutchinson MR, Gentgall M, Rolan P (2014). Reduced response to the thermal grill illusion in chronic pain patients. Pain Med..

[27] Staud R, Rodriguez ME (2006). Mechanisms of disease: Pain in fibromyalgia syndrome. Nat. Clin. Pract. Rheumatol..

[28] O'Brien AT, Deitos A, Pego YT, Fregni F, Carrillo-de-la-Peña MT (2018). Defective endogenous pain modulation in fibromyalgia: A meta-analysis of temporal summation and conditioned pain modulation paradigms. J. Pain.

[29] Clauw DJ (2014). Fibromyalgia: A clinical review. JAMA.

[30] Deutsche Schmerzgesellschaft (DGSS). AWMF-leitlinie-definition, pathophysiologie, diagnostik und therapie des fibromyalgiesyndroms, https://www.awmf.org/uploads/tx_szleitlinien/145-004l_S3_Fibromyalgiesyndrom_2019-11_1.pdf (2017).

[31] Wolfe F (2016). 2016 Revisions to the 2010/2011 fibromyalgia diagnostic criteria. Semin. Arthritis. Rheum..

[32] Martínez-Lavín M (2018). Fibromyalgia and small fiber neuropathy: the plot thickens!. Clin. Rheumatol..

[33] Lindstedt F (2011). Evidence for thalamic involvement in the thermal grill illusion: An FMRI study. PLoS ONE.

[34] Adam F (2023). Thermal grill illusion of pain in patients with chronic pain: a clinical marker of central sensitization?. Pain.

[35] Herrero JF, Laird JM, Lopez-Garcia JA (2000). Wind-up of spinal cord neurones and pain sensation: much ado about something?. Prog. Neurobiol..

[36] Staud R, Vierck CJ, Cannon RL, Mauderli AP, Price DD (2001). Abnormal sensitization and temporal summation of second pain (wind-up) in patients with fibromyalgia syndrome. Pain.

[37] Bourke JH (2021). Central sensitisation in chronic fatigue syndrome and fibromyalgia; a case control study. J. Psychosom. Res..

[38] Szikszay TM, Lévénez JLM, von Selle J, Adamczyk WM, Luedtke K (2021). Investigation of correlations between pain modulation paradigms. Pain Med. (Malden, Mass).

[39] Harper DE, Hollins M (2014). Coolness both underlies and protects against the painfulness of the thermal grill illusion. Pain.

[40] Ran C, Hoon MA, Chen X (2016). The coding of cutaneous temperature in the spinal cord. Nat Neurosci.

[41] Baron, R. 5.57-Neuropathic pain: Cinical in the senses: A comprehensive reference (eds. Masland, R. H. et al.) (Elsevier Inc., 2008).

[42] Ochoa JL, Yarnitsky D (1994). The triple cold syndrome. Cold hyperalgesia, cold hypoaesthesia and cold skin in peripheral nerve disease. Brain : J. Neurol..

[43] Gracely RH (2004). Pain catastrophizing and neural responses to pain among persons with fibromyalgia. Brain : J. Neurol..

[44] Scheuren R, Sütterlin S, Anton F (2014). Rumination and interoceptive accuracy predict the occurrence of the thermal grill illusion of pain. BMC Psychol..

[45] Bräscher AK, Sütterlin S, Scheuren R, Van den Bergh O, Witthöft M (2020). Somatic symptom perception from a predictive processing perspective: An empirical test using the thermal grill illusion. Psychosom. Med..

[46] Boettger MK, Schwier C, Bär KJ (2011). Sad mood increases pain sensitivity upon thermal grill illusion stimulation: Implications for central pain processing. Pain.

[47] Dubner R, Ren K, Gebhart GF, Schmidt RF (2013). Descending modulation of nociceptive processing. Encyclopedia of pain.

[48] Harper DE, Hollins M (2017). Conditioned pain modulation dampens the thermal grill illusion. Eur. J. Pain.

[49] Nahman-Averbuch H, Nir RR, Sprecher E, Yarnitsky D (2016). Psychological factors and conditioned pain modulation: A meta-analysis. Clin. J. Pain.

[50] Traxler J, Hanssen MM, Lautenbacher S, Ottawa F, Peters ML (2019). General versus pain-specific cognitions: Pain catastrophizing but not optimism influences conditioned pain modulation. Eur. J. Pain.

[51] Paricio-Montesinos R (2020). The sensory coding of warm perception. Neuron.

[52] Oudejans L (2016). Cornea nerve fiber quantification and construction of phenotypes in patients with fibromyalgia. Sci. Rep..

[53] Uceyler N (2013). Small fibre pathology in patients with fibromyalgia syndrome. Brain : J. Neurol..

[54] Rehm S (2021). Pain matters for central sensitization: sensory and psychological parameters in patients with fibromyalgia syndrome. Pain Rep..

[55] Kingdom F, Prins N (2010). Psychophysics: A practical introduction.

[56] Rolke R (2006). Quantitative sensory testing in the German research network on neuropathic pain (DFNS): Standardized protocol and reference values. Pain.

[57] Macfarlane GJ (2017). EULAR revised recommendations for the management of fibromyalgia. Ann. Rheum. Dis..

[58] Rolke R (2006). Quantitative sensory testing: A comprehensive protocol for clinical trials. Eur. J. Pain.

[59] Nilges P, Essau C (2015). Depression, anxiety and stress scales: DASS–A screening procedure not only for pain patients. Schmerz (Berlin, Germany).

[60] Frettloh J, Maier C, Gockel H, Huppe M (2003). Validation of the German mainz pain staging system in different pain syndromes. Schmerz (Berlin, Germany).

[61] Schisterman EF, Cole SR, Platt RW (2009). Overadjustment bias and unnecessary adjustment in epidemiologic studies. Epidemiology.

[62] The World Medical Association. Declaration of Helsinki-updated version Seoul, Korea 2008, htps://www.wma.net/what-we-do/medical-ethics/declaration-of-helsinki/doh-oct2008/ (2008).

