# Supplementary material for: Strong and aversive cold processing and pain facilitation in fibromyalgia patients relates to augmented thermal grill illusion
Source: Sci Rep. 2023 Sep 25;13:15982. doi: 10.1038/s41598-023-42288-7 (PMC10520026; doi:10.1038/s41598-023-42288-7)
Supplement: Supplementary file 1 — Supplementary Tables. [file 41598_2023_42288_MOESM1_ESM.pdf]

Supplementary tables

Strong and aversive cold processing and pain facilitation in fibromyalgia patients relates to augmented thermal grill illusion

Petra Bäumler, Anna Brenske, Andreas Winkelmann, Dominik Irnich, Beate Averbeck

Supplementary Table S1: Associations between thermal grill-evoked sensations and quantitative sensory testing (QST) among fibromyalgia patients

	Thermal grill (20°C /40°C)-evoked sensations											
	**cold** NRS (0 - 100) n=40		**warm** NRS (0 - 100) n=40		**unpleasantness** NRS (0 - 100) n=40		**pain** yes n=15 / no n=25		**pain intensity if pain > 0** NRS (0 - 100) n=25		**burning** yes n=15 / no n=25	
	crude β	adjusted β	crude β	adjusted β	crude β	adjusted β	crude OR	adjusted OR	crude β	adjusted β	crude OR	adjusted OR
**CDT$_{log}$**	10.77	25.41	8.49	-1.21	26.97	22.65	1.07	0.54	29.09	37.34	0.11	0.02
Δ °C from 32°C	[-21.54; 43.09]	[-6.75; 57.58]	[-21.22; 38.19]	[-33.25; 30.83]	[-17.41; 71.36]	[-25.78; 71.08]	[0.03; 40.24]	[0.01; 44.79]	[-11.55; 69.72]	[-9.20; 83.87]	[0.00; 4.81]	[0.00; 2.59]
	p = 0.514	p = 0.121	p = 0.575	p = 0.941	p = 0.234	p = 0.359	p = 0.970	p = 0.784	p = 0.161	p = 0.116	p = 0.255	p = 0.113
**WDT$_{log}$**	12.32	5.53	24.18	10.70	22.68	14.79	2.93	1.70	**55.75**	50.53	0.67	0.32
Δ °C from 32°C	[-25.91; 50.56]	[-30.71; 41.76]	[-10.29; 58.65]	[-24.24; 45.65]	[-30.29; 75.64]	[-38.62; 68.20]	[0.04; 239.54]	[0.01; 237.58]	**[6.70; 104.79]**	[-0.63; 101.68]	[0.01; 47.98]	[0.00; 69.81]
	p = 0.528	p = 0.765	p = 0.169	p = 0.548	p = 0.401	p = 0.587	p = 0.632	p = 0.834	**p = 0.026***	p = 0.053	p = 0.856	p = 0.681
**TSL$_{log}$**	26.79	22.32	5.43	-0.10	27.28	31.54	10.41	38.39	12.09	13.57	0.79	0.57
Δ °C CDT / WDT	[-3.50; 57.09]	[-6.24; 50.88]	[-23.36; 34.21]	[-28.53; 28.33]	[-15.56; 70.13]	[-10.77; 73.85]	[0.27; 396.60]	[0.37; 3944.57]	[-34.17; 58.34]	[-29.44; 56.57]	[0.02; 26.56]	[0.01; 38.01]
	p = 0.083	p = 0.126	p = 0.712	p = 0.995	p = 0.212	p = 0.144	p = 0.207	p = 0.123	p = 0.609	p = 0.536	p = 0.895	p = 0.794
**CPT**	**0.81**	**0.67**	0.50	0.30	0.54	0.32	1.08	1.10	-0.04	-0.13	1.03	1.00
°C	**[0.14; 1.47]**	**[0.02; 1.32]**	[-0.13; 1.13]	[-0.36; 0.95]	[-0.43; 1.51]	[-0.69; 1.32]	[0.99; 1.17]	[1.00; 1.21]	[-1.11; 1.03]	[-1.20; 0.95]	[0.95; 1.12]	[0.91; 1.10]
	**p = 0.017***	**p = 0.044***	p = 0.121	p = 0.376	p = 0.278	p = 0.535	p = 0.073	p = 0.061	p = 0.945	p = 0.819	p = 0.447	p = 1.000
**HPT**	-0.33	-0.44	0.54	0.57	-0.46	-0.34	0.98	0.98	0.06	0.11	0.95	0.99
°C	[-1.80; 1.13]	[-1.75; 0.87]	[-0.80; 1.87]	[-0.70; 1.83]	[-2.50; 1.57]	[-2.28; 1.61]	[0.83; 1.15]	[0.82; 1.17]	[-1.92; 2.04]	[-1.85; 2.07]	[0.81; 1.12]	[0.81; 1.20]
	p = 0.657	p = 0.509	p = 0.432	p = 0.380	p = 0.656	p = 0.733	p = 0.787	p = 0.847	p = 0.952	p = 0.913	p = 0.562	p = 0.902
**MPT$_{log}$**	-10.39	-12.79	-11.26	-10.55	-15.39	-15.87	0.13	0.13	-11.45	-15.61	1.09	1.12
mN	[-30.62; 9.83]	[-30.18; 4.60]	[-29.73; 7.22]	[-27.50; 6.39]	[-43.46; 12.68]	[-41.77; 10.03]	[0.01; 1.79]	[0.01; 1.96]	[-39.65; 16.75]	[-40.80; 9.57]	[0.11; 10.61]	[0.10; 13.08]
	p = 0.314	p = 0.149	p = 0.232	p = 0.222	p = 0.283	p = 0.230	p = 0.127	p = 0.139	p = 0.426	p = 0.224	p = 0.944	p = 0.929
**MPS$_{log}$**	0.14	1.34	2.95	2.80	4.90	6.32	2.29	2.83	4.80	4.79	0.85	1.12
NRS 0-100	[-9.96; 10.24]	[-7.71; 10.38]	[-6.28; 12.18]	[-5.92; 11.51]	[-9.06; 18.86]	[-6.91; 19.55]	[0.67; 7.79]	[0.70; 11.46]	[-10.23; 19.83]	[-8.67; 18.25]	[0.27; 2.62]	[0.28; 4.56]
	p = 0.978	p = 0.772	p = 0.531	p = 0.529	p = 0.492	p = 0.349	p = 0.184	p = 0.146	p = 0.532	p = 0.486	p = 0.775	p = 0.869
**TS**	11.34	12.78	**20.78**	**18.43**	**29.42**	**30.25**	**57.31**	**54.43**	-4.69	-8.67	17.52	25.89
WUR$_{log}$	[-10.45; 33.13]	[-6.51; 32.07]	**[1.57; 39.99]**	**[0.24; 36.61]**	**[0.12; 58.72]**	**[2.69; 57.80]**	**[1.86; 1764.41]**	**[1.42; 2085.68]**	[-42.64; 33.25]	[-44.07; 26.72]	[0.86; 355.95]	[0.83; 805.81]
	p = 0.308	p = 0.194	**p = 0.034***	**p = 0.047***	**p = 0.049***	**p = 0.031***	**p = 0.021***	**p = 0.032***	p = 0.808	p = 0.631	p = 0.062	p = 0.064
**PPT$_{log}$**	-38.49	-34.50	4.31	11.32	-23.54	-40.83	0.18	0.02	-6.23	0.33	0.09	0.19
kPa	[-78.08; 1.10]	[-77.62; 8.62]	[-33.63; 42.24]	[-31.53; 54.16]	[-80.56; 33.48]	[-105.25; 23.59]	[0.00; 18.70]	[0.00; 9.26]	[-65.80; 53.34]	[-63.38; 64.04]	[0.00; 10.25]	[0.00; 97.51]
	p = 0.057	p = 0.117	p = 0.824	p = 0.605	p = 0.418	p = 0.214	p = 0.465	p = 0.212	p = 0.838	p = 0.992	p = 0.318	p = 0.601
**MDT$_{log}$**	-6.54	-7.23	-9.00	**-14.98**	-7.30	-12.00	0.47	0.27	14.50	13.69	1.38	1.46
mN	[-24.10; 11.01]	[-23.00; 8.53]	[-24.98; 6.98]	**[-29.67; -0.29]**	[-31.77; 17.16]	[-35.24; 11.23]	[0.06; 3.47]	[0.03; 2.71]	[-12.93; 41.93]	[-12.25; 39.63]	[0.19; 10.07]	[0.15; 14.77]
	p = 0.465	p = 0.369	p = 0.269	**p = 0.046***	p = 0.558	p = 0.311	p = 0.460	p = 0.268	p = 0.300	p = 0.301	p = 0.754	p = 0.746
**VDT Limit**	-1.40	-1.84	**-17.25**	**-13.85**	-6.69	-2.55	0.80	1.40	-14.16	-10.02	0.49	0.68
x / 8	[-15.27; 12.47]	[-14.63; 10.94]	**[-28.81; -5.68]**	**[-25.47; -2.23]**	[-25.87; 12.49]	[-21.45; 16.35]	[0.17; 3.83]	[0.24; 8.17]	[-34.31; 5.99]	[-28.74; 8.69]	[0.10; 2.51]	[0.10; 4.46]
	p = 0.843	p = 0.778	**p = 0.003***	**p = 0.019***	p = 0.494	p = 0.791	p = 0.780	p = 0.706	p = 0.168	p = 0.294	p = 0.394	p = 0.684
**VDT Level$_{log}$**	7.85	4.29	13.29	6.53	15.85	6.20	1.79	1.20	**18.48**	8.88	2.76	2.14
microns	[-8.07; 23.76]	[-11.41; 19.99]	[-0.90; 27.49]	[-8.59; 21.64]	[-5.99; 37.70]	[-17.01; 29.41]	[0.29; 11.05]	[0.14; 10.00]	**[0.11; 36.85]**	[-12.04; 29.80]	[0.42; 18.21]	[0.25; 18.20]
	p = 0.334	p = 0.592	p = 0.067	p = 0.397	p = 0.155	p = 0.600	p = 0.532	p = 0.868	**p = 0.049***	p = 0.405	p = 0.291	p = 0.487
**DMA**	1.15	-0.27	0.71	-1.80	6.36	4.50	2.53	2.94	-1.47	-3.92	1.26	1.28
yes n=9	[-12.59; 14.89]	[-12.58; 12.05]	[-11.91; 13.33]	[-13.70; 10.11]	[-12.65; 25.37]	[-13.65; 22.64]	[0.45; 14.20]	[0.45; 19.29]	[-19.93; 17.00]	[-21.24; 13.41]	[0.26; 6.03]	[0.22; 7.50]
no n= 31	p = 0.870	p = 0.966	p = 0.912	p = 0.767	p = 0.512	p = 0.627	p = 0.292	p = 0.261	p = 0.876	p = 0.658	p = 0.770	p = 0.787

Regression coefficients (β) and odds ratios (OR) with 95% confidence intervals according to generalized linear models (GLM); Adjustment for age, pain during the last week before inclusion and DASS scores for depression, anxiety and stress; Fisher test in case of mutual exclusive categories of the dependent and independent variable without adjustment; *: associations significant on an α-level of 5%; CDT: cold detection threshold as change from 32 °C (Δ °C); WDT: warm detection threshold as change from 32 °C (Δ °C); TSL: thermal sensory limen as temperature change between cold and warm detection (Δ °C CDT/WDT); CPT: cold pain threshold in °C; HPT: heat pain threshold in °C; MDT: mechanical detection threshold in millinewton (mN); MPT: mechanical pain threshold in mN; MPS: mechanical pain sensitivity on the numeric rating scale (NRS 0-100); TS: temporal summation as evaluated by the wind-up ratio (WUR); VDT_{Lim}: vibration detection threshold by the method of limits (x/8); VDT_{Lev}: vibration detection threshold by the method of levels in microns; PPT: pressure pain threshold in kilopascal (kPa)

Supplementary Table S2: Associations of thermal grill-evoked sensations with quantitative sensory testing as well as with cold- and warm-evoked sensations in healthy volunteers

	Thermal grill (20°C /40°C)-evoked sensations			
	**cold**<br>NRS (0 - 100) n=20	**warm**<br>NRS (0 - 100) n=20	**unpleasantness**<br>NRS (0 - 100) n=20	**burning**<br>yes n=11 / no n=9
	crude β	crude β	crude β	crude OR
**CDT_{log}**<br>Δ °C from 32°C	-16.79<br>[-48.82; 15.24]<br>p = 0.304	-9.93<br>[-38.10; 18.23]<br>p = 0.489	30.80<br>[-11.81; 73.41]<br>p = 0.157	0.00<br>[0.00; 3.08]<br>p = 0.091
**WDT_{log}**<br>Δ °C from 32°C	-42.33<br>[-85.54; 0.88]<br>p = 0.055	**-44.01**<br>**[-79.93; -8.09]**<br>**p = 0.016***	4.99<br>[-58.93; 68.90]<br>p = 0.878	0.00<br>[0.00; 1.17]<br>p = 0.052
**TSL_{log}**<br>Δ °C CDT / WDT	-11.85<br>[-48.70; 25.01]<br>p = 0.529	-6.25<br>[-38.41; 25.90]<br>p = 0.703	-10.82<br>[-61.21; 39.57]<br>p = 0.674	0.58<br>[0.00; 415.85]<br>p = 0.870
**CPT**<br>°C	-0.59<br>[-1.32; 0.14]<br>p = 0.116	-0.04<br>[-0.71; 0.63]<br>p = 0.911	after removal of 2 outliers<br>-0.36 [-1,31; 0.60]<br>p = 0.464	1.18<br>[0.98; 1.42]<br>p = 0.077
**HPT**<br>°C	-1.51<br>[-3.41; 0.40]<br>p = 0.121	-1.07<br>[-2.76; 0.61]<br>p = 0.213	-0.41<br>[-3.15; 2.33]<br>p = 0.768	0.98<br>[0.69; 1.40]<br>p = 0.919
**MPT_{log}**<br>mN	2.92<br>[-12.73; 18.58]<br>p = 0.714	7.50<br>[-5.71; 20.72]<br>p = 0.266	5.40<br>[-15.83; 26.63]<br>p = 0.618	2.30<br>[0.13; 39.10]<br>p = 0.566
**MPS_{log}**<br>NRS (0-100)	7.23<br>[-4.74; 19.21]<br>p = 0.237	7.55<br>[-2.68; 17.77]<br>p = 0.148	8.71<br>[-7.71; 25.12]<br>p = 0.299	2.26<br>[0.23; 21.69]<br>p = 0.481
**TS**<br>WUR_{log}	-22.75<br>[-50.47; 4.96]<br>p = 0.108	-8.27<br>[-33.55; 17.02]<br>p = 0.522	**-43.31**<br>**[-78.59; -8.02]**<br>**p = 0.016***	2.78<br>[0.01; 552.61]<br>p = 0.705
**PPT_{log}**<br>kPa	-3.69<br>[-63.18; 55.79]<br>p = 0.903	-10.69<br>[-62.08; 40.69]<br>p = 0.683	43.02<br>[-35.69; 121.73]<br>p = 0.284	**$6.03*10^{-8}$**<br>**[$5.62*10^{-15}$; 0.65]**<br>**p = 0.044***
**MDT_{log}**<br>mN	-2.21<br>[-26.39; 21.97]<br>p = 0.858	-16.63<br>[-36.31; 3.04]<br>p = 0.098	-14.04<br>[-46.38; 18.30]<br>p = 0.395	0.20<br>[0.00; 15.94]<br>p = 0.472
**VDT Limit**<br>x / 8	-11.08<br>[-48.49; 26.34]<br>p = 0.562	-2.81<br>[-35.51; 29.88]<br>p = 0.866	-8.60<br>[-59.78; 42.58]<br>p = 0.742	0.75<br>[0.00; 613.91]<br>p = 0.932
**VDT $Level_{log}$**<br>microns	-6.71<br>[-16.78; 3.36]<br>p = 0.191	-6.04<br>[-14.73; 2.64]<br>p = 0.172	3.70<br>[-10.66; 18.06]<br>p = 0.613	0.19<br>[0.02; 2.08]<br>p = 0.173
**20°-evoked cold**<br>NRS (0 - 100)	**0.82**<br>**[0.37; 1.27]**<br>**p < 0.001***	**0.55**<br>**[0.12; 0.99]**<br>**p = 0.013***	0.49<br>[-0.26; 1.24]<br>p = 0.201	1.02<br>[0.92; 1.13]<br>p = 0.642
**40°-evoked warm**<br>NRS (0 - 100)	**0.86**<br>**[0.42; 1.29]**<br>**p < 0.001***	**0.64**<br>**[0.23; 1.06]**<br>**p = 0.002***	0.64<br>[-0.09; 1.37]<br>p = 0.084	1.00<br>[0.91; 1.11]<br>p = 0.955

Regression coefficients (β) and odds ratios (OR) with 95% confidence intervals according to generalized linear models (GLM); ; *: associations significant on an α-level of 5%;); CDT: cold detection threshold as change from 32 °C (Δ °C); WDT: warm detection threshold as change from 32 °C (Δ °C); TSL: thermal sensory limen as temperature change between cold and warm detection (Δ °C CDT/WDT); CPT: cold pain threshold in °C; HPT: heat pain threshold in °C; MDT: mechanical detection threshold in millinewton (mN); MPT: mechanical pain threshold in mN; MPS: mechanical pain sensitivity on the numeric rating scale (NRS 0-100); TS: temporal summation as evaluated by the wind-up ratio (WUR); VDT_{Lim}: vibration detection threshold by the method of limits (x/8); VDT_{Lev}: vibration detection threshold by the method of levels in microns; PPT: pressure pain threshold in kilopascal (kPa); NRS: numeric rating scale

Supplementary Table S3: Associations between thermal grill-evoked sensations and sensations evoked by the 20°C- and 40°C-thermal plate in fibromyalgia patients

	**Thermal grill (20°C /40°C)-evoked sensations**											
	**cold**<br>NRS (0 - 100) n=40		**warm**<br>NRS (0 - 100) n=40		**unpleasantness**<br>NRS (0 - 100) n=40		**pain**<br>yes n=25 / no n=15		**pain intensity if pain > 0**<br>NRS (0 - 100) n=25		**burning**<br>yes n=15 / no n=25	
	crude β	adjusted β	crude β	adjusted β	crude β	adjusted β	crude OR	adjusted OR	crude β	adjusted β	crude OR	adjusted OR
**20°-evoked sensations**												
**cold**<br>NRS (0 - 100)<br>n=40	**0.88**<br>**[0.62; 1.15]**<br>**p < 0.001***	**0.77**<br>**[0.49; 1.05]**<br>**p < 0.001***	**0.65**<br>**[0.36; 0.93]**<br>**p < 0.001***	**0.68**<br>**[0.39; 0.96]**<br>**p < 0.001***	**1.11**<br>**[0.71; 1.51]**<br>**p < 0.001***	**1.21**<br>**[0.82; 1.60]**<br>**p < 0.001***	1.04<br>[0.99; 1.09]<br>p = 0.130	1.05<br>[0.99; 1.11]<br>p = 0.084	**0.75**<br>**[0.25; 1.24]**<br>**p = 0.003***	**0.77**<br>**[0.27; 1.26]**<br>**p = 0.002***	1.03<br>[0.99; 1.08]<br>p = 0.153	1.02<br>[0.97; 1.08]<br>p = 0.374
**unpleasantness**<br>yes n=26<br>no n=14	**19.45**<br>**[9.03; 29.87]**<br>**p < 0.001***	**13.09**<br>**[0.40; 25.77]**<br>**p = 0.043***	**11.93**<br>**[1.52; 22.34]**<br>**p = 0.025***	11.80<br>[-0.55; 24.16]<br>p = 0.061	**25.32**<br>**[10.54; 40.10]**<br>**p = 0.001***	**27.97**<br>**[10.29; 45.64]**<br>**p = 0.002***	**6.00**<br>**[1.44; 24.92]**<br>**p = 0.014***	**188.28**<br>**[6.04; 5870.41]**<br>**p = 0.003***	13.58<br>[-6.46; 33.62]<br>p = 0.184	4.83<br>[-19.57; 29.23]<br>p = 0.698	**10.50**<br>**[2.31; 47.78]**<br>**p = 0.002***	**20.00**<br>**[1.81; 221.38]**<br>**p = 0.015***
**unpleasantness**<br>**if > 0** n=26<br>NRS (0 - 100)	0.32<br>[-0.16; 0.80]<br>p = 0.193	-	0.07<br>[-0.39; 0.53]<br>p = 0.762	-	0.42<br>[-0.22; 1.06]<br>p = 0.198	-	0.98<br>[1.05; 0.92]<br>p = 0.634	-	0.71<br>[-0.10; 1.52]<br>p = 0.086	-	**0.89**<br>**[0.98; 0.80]**<br>**p = 0.020***	-
**pain**<br>yes n=10<br>no n=30	3.11<br>[-10.11; 16.33]<br>p = 0.645	-1.24<br>[-13.87; 11.38]<br>p = 0.847	5.28<br>[-6.78; 17.34]<br>p = 0.391	2.35<br>[-9.85; 14.55]<br>p = 0.706	0.72<br>[-17.71; 19.15]<br>p = 0.939	0.36<br>[-18.30; 19.03]<br>p = 0.970	**Fisher-Test**<br>**p = 0.006***	*All with 20°C-evoked pain also thermal grill-evoked pain-*	-3.67<br>[-20.54; 13.20]<br>p = 0.670	-6.63<br>[-24.39; 11.13]<br>p = 0.465	3.06<br>[0.55; 16.90]<br>p = 0.200	2.13<br>[0.32; 14.30]<br>p = 0.436
**pain if > 0**<br>n=10<br>NRS (0 - 100)	-0.20<br>[-1.21; 0.82]<br>p = 0.705	-	0.03<br>[-0.72; 0.78]<br>p = 0.937	-	-0.49<br>[-1.54; 0.57]<br>p = 0.365	-	-	-	-0.06<br>[-1.16; 1.05]<br>p = 0.921	-	-0.92<br>[0.79; 1.08]<br>p = 0.304	-
**burning**<br>yes n=8<br>no n=32	6.41<br>[-7.81; 20.62]<br>p = 0.377	2.96<br>[-13.30; 19.22]<br>p = 0.721	**15.42**<br>**[3.14; 27.69]**<br>**p = 0.014***	13.35<br>[-1.86; 28.56]<br>p = 0.085	10.68<br>[-9.00; 30.35]<br>p = 0.287	11.40<br>[-12.41; 35.21]<br>p = 0.348	5.44<br>[0.60; 49.56]<br>p = 0.133	7.60<br>[0.39; 146.72]<br>p = 0.179	7.46<br>[-10.78; 25.70]<br>p = 0.423	4.04<br>[-18.19; 26.27]<br>p = 0.722	**Fisher-Test**<br>**p = 0.016***	*All with 20°C-evoked burning also thermal grill-evoked burning*
**40°C-evoked sensations**												
**warm**<br>NRS (0 - 100)<br>n=40	**0.68**<br>**[0.26; 1.10]**<br>**p = 0.002***	**0.68**<br>**[0.31; 1.05]**<br>**p < 0.001***	0.34<br>[-0.08; 0.76]<br>p = 0.118	0.27<br>[-0.13; 0.68]<br>p = 0.186	0.56<br>[-0.07; 1.19]<br>p = 0.083	0.36<br>[-0.26; 0.98]<br>p = 0.253	1.00<br>[0.95; 1.05]<br>p = 0.996	1.00<br>[0.94; 1.06]<br>p = 0.895	0.61<br>[-0.10; 1.31]<br>p = 0.091	0.55<br>[-0.12; 1.22]<br>p = 0.107	1.03<br>[0.97; 1.08]<br>p = 0.373	1.02<br>[0.96; 1.08]<br>p = 0.585
**unpleasantness**<br>yes n=12<br>no n=28	0.08<br>[-12.45; 12.61]<br>p = 0.990	-3.53<br>[-16.36; 9.31]<br>p = 0.590	-3.12<br>[-14.57; 8.34]<br>p = 0.594	-7.98<br>[-20.20; 4.24]<br>p = 0.201	0.10<br>[-17.32; 17.51]<br>p = 0.991	-10.53<br>[-29.29; 8.23]<br>p = 0.271	1.29<br>[0.31; 5.35]<br>p = 0.722	1.88<br>[0.30; 11.89]<br>p = 0.504	2.03<br>[-15.73; 19.80]<br>p = 0.822	-9.28<br>[-27.94; 9.39]<br>p = 0.330	2.25<br>[0.50; 10.14]<br>p = 0.291	3.32<br>[0.48; 22.91]<br>p = 0.223
**unpleasantness**<br>**if > 0** n=12<br>NRS (0 - 100)	**0.73**<br>**[0.01; 1.44]**<br>**p = 0.047***	-	0.65<br>[-0.03; 1.32]<br>p = 0.061	-	**1.29**<br>**[0.48; 2.10]**<br>**p = 0.002***	-	1.02<br>[0.92; 1.12]<br>p = 0.765	-	**1.19**<br>**[0.57; 1.81]**<br>**p < 0.001***	-	1.07<br>[0.93; 1.22]<br>p = 0.359	-
**burning**<br>yes n=25<br>no n=15	7.15<br>[-5.52; 19.81]<br>p = 0.269	5.11<br>[-6.60; 16.81]<br>p = 0.393	2.20<br>[-9.58; 13.98]<br>p = 0.714	-2.93<br>[-14.32; 8.47]<br>p = 0.615	8.33<br>[-9.35; 26.02]<br>p = 0.356	1.34<br>[-16.11; 18.79]<br>p = 0.880	1.88<br>[0.41; 8.60]<br>p = 0.414	1.46<br>[0.28; 7.60]<br>p = 0.654	2.95<br>[-14.79; 20.70]<br>p = 0.744	-4.05<br>[-21.26; 13.16]<br>p = 0.645	**9.33**<br>**[1.05; 82.64]**<br>**p = 0.045***	7.82<br>[0.77; 79.80]<br>p = 0.083

Regression coefficients (β) and odds ratios (OR) with 95% confidence intervals according to generalized linear models (GLM); Adjustment for age, pain during the last week before inclusion and DASS scores for depression, anxiety and stress; Fisher test in case of mutual exclusive categories of the dependent and independent variable without adjustment; *: associations significant on an α-level of 5%; NRS: numeric rating scale

Supplementary Table S4: Associations between thermal detection / pain thresholds and cold (20°C)- / warm (40°C)-evoked sensations in fibromyalgia patients

	**Fibromyalgia patients – cold (20°C)-evoked sensations**					**Healthy controls – cold (20°C)- evoked cold sensation**<br>NRS (0 – 100)
	**cold**<br>NRS (0 - 100)	**unpleasantness**<br>yes / no	**unpleasantness if > 0**<br>NRS (0 - 100)	**pain**<br>yes /no	**burning**<br>yes / no	
	β	OR	β	OR	OR	β
**CDT$_{log}$**<br>Δ °C	16.25<br>[-9.74; 42.25]<br>p = 0.220	0.07<br>[0.00; 3.39]<br>p = 0.180	21.09<br>[-8.09; 50.26]<br>p = 0.157	7.88<br>[0.11; 584.28]<br>p = 0.347	11.73<br>[0.10; 1356.91]<br>p = 0.310	-0.15<br>[-25.21; 24.92]<br>p = 0.991
**CPT**<br>°C	0.44<br>[-0.12; 1.00]<br>p = 0.120	**1.10**<br>**[1.01; 1.21]**<br>**p = 0.030***	-0.11<br>[-0.77; 0.55]<br>p = 0.744	0.91<br>[0.81; 1.02]<br>p = 0.100	0.98<br>[0.88; 1.08]<br>p = 0.651	0.13<br>[-0.46; 0.72]<br>p = 0.658
	**Fibromyalgia patients – warm (40°C)-evoked sensations**					**Healthy controls – warm (40°C)-evoked warm sensation**<br>NRS (0 – 100)
	**warm**<br>NRS (0 – 100)	**unpleasantness**<br>yes / no	**unpleasantness if > 0**<br>NRS (0 - 100)	**pain**<br>yes /no	**burning**<br>yes / no	
	β	OR	β	OR	OR	β
**WDT$_{log}$**<br>Δ °C	-9.04<br>[-34.12; 16.03]<br>p = 0.480	8.24<br>[0.06; 1138.18]<br>p = 0.402	13.62<br>[-40.68; 67.91]<br>p = 0.623	0.01<br>[0.00; 7.14]<br>p = 0.157	1.12<br>[0.01; 118.37]<br>p = 0.962	-8.73<br>[-44.33; 26.87]<br>p = 0.631
**HPT**<br>°C	**-1.11**<br>**[-2.01; -0.21]**<br>**p = 0.016***	1.03<br>[0.87; 1.23]<br>p = 0.734	-0.91<br>[-2.44; 0.61]<br>p = 0.241	0.92<br>[0.68; 1.23]<br>p = 0.567	1.11<br>[0.92; 1.35]<br>p = 0.268	**-1.66**<br>**[-3.02; -0.31]**<br>**p = 0.016***

Regression coefficients (β) and odds ratios (OR) with 95% confidence intervals according to generalized linear models (GLM); *: associations significant on an α-level of 5%; CDT: cold detection threshold as change from 32°C (Δ °C); WDT: warm detection threshold as change form 32 °C (Δ °C); CPT: cold pain threshold in °C; HPT: heat pain threshold in °C; NRS: numeric rating scale

Supplementary Table S5: Independent association of strong cold-evoked sensations and high temporal summation (TS) with the thermal grill illusion in fibromyalgia patients

	**Thermal grill (20°C /40°C)-evoked sensations**											
	**cold**<br>NRS (0 - 100) n=40		**warm**<br>NRS (0 - 100) n=40		**unpleasantness**<br>NRS (0 - 100) n=40		**pain**<br>yes n=15 / no n=25		**pain if pain > 0**<br>NRS (0 - 100) n=25		**burning**<br>yes n=15 / no n=25	
	crude β	adjusted β	crude β	adjusted β	crude β	adjusted β	crude OR	adjusted OR	crude β	adjusted β	crude OR	adjusted OR
**20°C-evoked cold**<br>NRS (0 - 100)	**0.87**<br>**[0.60; 1.14]**<br>**p < 0.001***	**0.75**<br>**[0.47; 1.03]**<br>**p < 0.001***	**0.61**<br>**[0.33; 0.88]**<br>**p < 0.001***	**0.64**<br>**[0.36; 0.92]**<br>**p < 0.001***	**1.06**<br>**[0.67; 1.45]**<br>**p < 0.001***	**1.15**<br>**[0.77; 1.53]**<br>**p < 0.001***	1.03<br>[0.98; 1.08]<br>p = 0.194	1.04<br>[0.98; 1.11]<br>p = 0.148	**0.74**<br>**[0.25; 1.24]**<br>**p = 0.003***	**0.76**<br>**[0.27; 1.25]**<br>**p = 0.002***	1.03<br>[0.98; 1.08]<br>p = 0.219	1.02<br>[0.96; 1.07]<br>p = 0.518
**TS**<br>WUR$_{log}$	3.59<br>[-11.91; 19.09]<br>p = 0.650	6.01<br>[-9.07; 21.10]<br>p = 0.435	15.37<br>[-0.70; 31.44]<br>p = 0.061	12.67<br>[-2.42; 27.75]<br>p = 0.100	20.01<br>[-2.71; 42.73]<br>p = 0.084	19.88<br>[-0.53; 40.30]<br>p = 0.056	**54.07**<br>**[1.65; 1770.77]**<br>**p = 0.025***	**47.18**<br>**[1.04; 2147.45]**<br>**p = 0.048***	-2.15<br>[-34.84; 30.54]<br>p = 0.898	-5.53<br>[-35.87; 24.82]<br>p = 0.721	15.68<br>[0.72; 342.71]<br>p = 0.080	23.22<br>[0.73; 738.75]<br>p = 0.075
**20°C-evoked unpleasantness**<br>yes n=26<br>no n=14	**18.95**<br>**[8.43; 29.46]**<br>**p < 0.001***	11.70<br>[-1.24; 24.63]<br>p = 0.076	**10.36**<br>**[0.24; 20.48]**<br>**p = 0.045***	9.38<br>[-2.94; 21.70]<br>p = 0.136	**23.32**<br>**[8.83; 37.81]**<br>**p = 0.002***	**24.50**<br>**[6.88; 42.11]**<br>**p = 0.006***	**6.14**<br>**[1.28; 29.53]**<br>**p = 0.024***	**79.37**<br>**[2.57; 2448.16]**<br>**p = 0.012***	13.50<br>[-6.54; 33.54]<br>p = 0.187	4.07<br>[-20.49; 28.62]<br>p = 0.745	**10.50**<br>**[2.12; 52.03]**<br>**p = 0.004***	**14.96**<br>**[1.48; 151.53]**<br>**p = 0.022***
**TS**<br>WUR$_{log}$	5.67<br>[-13.62; 24.95]<br>p = 0.565	8.69<br>[-10.43; 27.81]<br>p = 0.373	17.68<br>[-0.88; 36.24]<br>p = 0.062	15.14<br>[-3.07; 33.36]<br>p = 0.103	22.44<br>[-4.14; 49.01]<br>p = 0.098	21.67<br>[-4.37; 47.72]<br>p = 0.103	**64.40**<br>**[1.59; 2611.52]**<br>**p = 0.027***	15.26<br>[0.17; 1395.84]<br>p = 0.237	-3.66<br>[-40.39; 33.06]<br>p = 0.845	-7.84<br>[-43.52; 27.84]<br>p = 0.667	19.06<br>[0.58; 624.62]<br>p = 0.098	18.77<br>[0.38; 936.25]<br>p = 0.142

Regression coefficients (β) and odds ratios (OR) with 95% confidence intervals according to generalized linear models (GLM) with 20°C-evoked sensations and TS as independent variables; Adjustment for age, pain during the last week before inclusion and DASS scores for depression, anxiety and stress; *: associations significant on an α-level of 5%; TS: temporal summation as evaluated by the wind-up ratio (WUR); NRS: numeric rating scale
